# Electro‐Driven Multi‐Enzymatic Cascade Conversion of CO_2_ to Ethylene Glycol in Nano‐Reactor

**DOI:** 10.1002/advs.202407204

**Published:** 2024-09-04

**Authors:** Likun Luan, Yingfang Zhang, Xiuling Ji, Boxia Guo, Shaoyu Song, Yuhong Huang, Suojiang Zhang

**Affiliations:** ^1^ Beijing Key Laboratory of Ionic Liquids Clean Process CAS Key Laboratory of Green Process and Engineering State Key Laboratory of Mesoscience and Engineering Institute of Process Engineering Chinese Academy of Sciences Beijing 100190 P. R. China; ^2^ Sino‐Danish College University of Chinese Academy of Sciences Beijing 101408 P. R. China; ^3^ Longzihu New Energy Laboratory Zhengzhou Institute of Emerging Industrial Technology Henan University Zhengzhou 450000 P. R. China

**Keywords:** CO_2_ reduction, electro‐NADH regeneration, ethylene glycol, hydrogen‐bonded organic frameworks, multi‐enzymatic cascade conversion

## Abstract

Multi‐enzymatic cascade reaction provides a new avenue for C─C coupling directly from CO_2_ under mild conditions. In this study, a new pathway with four enzymes including formate dehydrogenase (PaFDH), formaldehyde dehydrogenase (BmFADH), glycolaldehyde synthase (PpGALS), and alcohol dehydrogenase (GoADH) is developed for directly converting CO_2_ gas molecules to ethylene glycol (EG) in vitro. A rhodium‐based NADH regeneration electrode is constructed to continuously provide the proton and electron of this multi‐enzymatic cascade reaction. The prepared electrode can reach the Faradaic Efficiency (FE) of 82.9% at −0.6 V (vs. Ag/AgCl) and the NADH productivity of 0.737 mM h^−1^. Shortening the reaction path is crucial for multi‐enzymatic cascade reactions. Here, a hydrogen‐bonded organic framework (HOF) nano‐reactor is successfully developed to immobilize four enzymes in one pot with a striking enzyme loading capacity (990 mg enzyme g^−1^ material). Through integrating and optimization of NADH electro‐regeneration and enzymatic catalysis in one pot, 0.15 mM EG is achieved with an average conversion rate of 7.15 × 10^−7^ mmol CO_2_ min^−1^ mg^−1^ enzymes in 6 h. These results shed light on electro‐driven multi‐enzymatic cascade conversion of C─C coupling from CO_2_ in the nano‐reactor.

## Graphical abstract

An electro‐driven enzymatic catalysis system is constructed to convert CO_2_ to value‐added products, for example, ethylene glycol (EG). The system is composed of rhodium‐grafted carbon fiber electrodes allowing NADH electro‐regeneration and hydrogen‐bonded organic frameworks (HOFs) confined enzymatic‐cascaded nanoreactor promoting substrate transfer. The system paves the way for CO_2_ “green” conversion to complex chemicals and products.

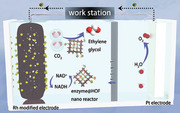

## 1 Introduction

Global rapid CO_2_ emissions have exhibited increasingly serious environmental problems. Sustainable conversion of CO_2_ to value‐added products such as fuels, bulk and commodity chemicals, sugars and starch, and even pharmaceuticals, paves the opportunities by exploiting CO_2_ feedstock instead of the fossil resource. However, CO_2_ conversion usually requires extra energy as its thermodynamically stable property (750 kJ mol^−1^ of C═O bond energy). Despite that electrocatalysis and biocatalysis offer the opportunity for C_1_ compound conversion as well as C─C coupling from CO_2_, CO_2_ reduction and utilization under mild conditions remains a great challenge. Currently, electrocatalysis can achieve extremely high current densities and very high selectivity for converting CO_2_ to C_1_ compounds, such as CO, HCOOH, and CH_4_. However, C_2+_ or C_3+_ products with higher energy density are more favored and more suitable as intermediates in the production of chemicals, such as ethylene, ethanol, ethylene glycol (EG), etc. Electro‐catalytical prolonging carbon chain through the addition of C_2+_ building blocks has suffered from low selectivity, low current density, and lack of efficient catalysts for the corresponding desired products.^[^ ^1^ ^]^ In contrast, biological reactions, characterized by mild conditions, high selectivity, and designable metabolic pathways, are now widely used for CO_2_ reduction, C─C coupling, and carbon chain extension. Ji et al.^[^ ^2^ ^]^ discovered a novel formate dehydrogenase (PaFDH) for CO_2_ bioactivation which could produce 0.42 ± 0.03 g L^−1^ formate in only 1 h in specific ionic liquid microenvironment. Dong et al.^[^ ^3^ ^]^ reported a complete anabolic pathway for the direct conversion of CO_2_ to ethanol by cascaded enzymes and obtained 0.37 mM at a conversion rate of 4.33 nmol CO_2_ min^−1^ mg^−1^ enzyme.

EG as a bulk chemical, has been extensively used for the production of biodegradable plastics such as polyethylene terephthalate (PET), polyglycolic acid (PGA), and polylactic‐co‐glycolic acid (PLGA).^[^ ^4^ ^]^ In addition, EG is also widely added in antifreeze reagents and coolants. Several in vitro enzymatic metabolic pathways for EG have been reported. Jo et al.^[^ ^5^ ^]^ coupled glyoxylate carboligase from *Escherichia coli* K‐12 (EcGCL) with lactaldehyde reductase (FucO) to produce 6.6 mM EG from formaldehyde and up to 66% bioconversion was reached. Tan et al.^[^ ^6^ ^]^ subjected methanol to multi‐enzymatic cascade pathways with alcohol oxidase (AOX), glycolaldehyde synthase (GALS), and alcohol dehydrogenases from *Gluconobacter oxydans* (GOX) and obtained 0.90 g L^−1^ EG. The use of formaldehyde and methanol as C_1_ compounds to synthesize EG has made great breakthroughs, however, the synthesis of EG using CO_2_ as the substrate under mild conditions by multi‐enzyme cascade reaction still has great challenges.

The main challenge for converting CO_2_ to EG by multi‐enzyme cascade reaction was the requirement of a high amount of the cofactor nicotinamide adenine dinucleotide (NADH), which provides the essential proton and electron. The simultaneous regeneration of NADH with a multi‐enzyme cascade reaction was highly in demand to improve the converting efficiency. NADH regeneration could not only cut the cost but also favor the continuation of multi‐enzyme cascade reaction with sufficient reducing equivalent. NADH regeneration has been realized by chemical,^[^ ^7^ ^]^ enzymatic,^[^ ^8^ ^]^ electrochemical,^[^ ^9^, ^10^ ^]^ and photochemical^[^ ^11^ ^]^ catalysis. Electrochemical catalysis, which provides mild conditions that match the enzyme reactions was far superior to other methods. Moreover, electrochemical NADH regeneration played a significant role in electro‐enzymatic catalysis system to fabricate a long‐time steady enzymatic catalysis system.^[^ ^12^ ^]^ The role of electricity tends to be either NADH regeneration^[^ ^13^ ^]^ or the use of other electron mediators to replace NADH and transfer electrons between the electrode and the enzyme.^[^ ^14^ ^]^ In view of this, combining multi‐enzymatic cascade reactions with electrocatalysis for NADH regeneration can make up for each other's shortcomings and amplify their respective flashpoints.

It is worth emphasizing that shortening the multi‐enzyme reaction route will be profitable to improve the electrical‐enzyme coupling system. Therefore, it is necessary to guarantee the mass transfer and the progress of the reaction in the proportional microenvironment/reactor of the cascade enzymes.^[^ ^15^ ^]^ In order to maximize the retention of enzyme activity and to match the enzyme size, porous materials capable of in situ encapsulation of enzymes under room temperature and aqueous‐phase conditions have been favored but are still in their early stages. The room‐temperature aqueous‐phase synthesized metal organic frameworks (MOFs) that have been reported for immobilizing enzymes are mainly bound to ZIF,^[^ ^16^ ^]^ which have narrow pores and limited mass transfer of enzymes.^[^ ^17^ ^]^ Most of the room‐temperature aqueous‐phase synthesized covalent organic framework (COF) materials use 1,3,5‐triformylbenzene (TB) or its modification as the precursor,^[^ ^8^, ^18^ ^]^ which is monotonous. While hydrogen‐bonded organic frameworks (HOFs) with unique hydrogen bonding between molecules and large aromatic rings have been discovered for in situ immobilizing enzymes by self‐assembling into crystal structures under ambient conditions.^[^ ^19^, ^20^ ^]^ The precursors usually contain aromatic rings with large conjugated regions containing carboxyl side chains,^[^ ^21^ ^]^ creating preferable conditions for immobilization of enzymes. It has also been reported that HOF nano‐reactor can modulate the metal coordination environment of enzyme active centers thereby altering their activities.^[^ ^22^ ^]^ Despite the successful trials for in situ immobilizing cytochrome c,^[^ ^22^ ^]^ catalase,^[^ ^23^ ^]^ and lipase,^[^ ^17^ ^]^ few attempts have been reported on multi‐enzymatic immobilization by HOF nano‐reactor.

**Scheme 1:**
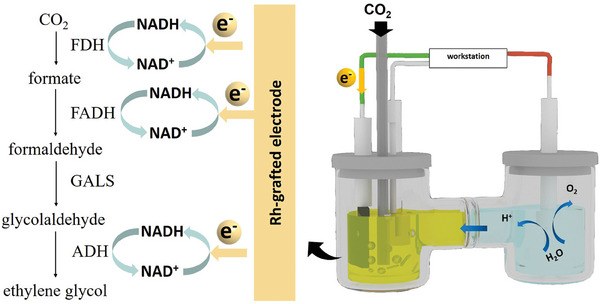
Illustration of electro‐enzymatic coupling system. Rh‐grafted carbon felt electrode provides the electrons for the regeneration of NADH, which provides the proton and electrons for the cascade enzyme reaction for converting CO_2_ to EG with PaFDH, BmFADH, PpGALS, and GoADH.

Herein, for the first time, we have demonstrated the feasibility of the multi‐enzymatic cascade reaction route for converting CO_2_ to EG with formate dehydrogenase from *Paracoccus sp*. MKU1 (PaFDH), formaldehyde dehydrogenase from *Burkholderia multivorans* (BmFADH), glycolaldehyde synthase from *Pseudomonas putida* (PpGALS), and alcohol dehydrogenases from *Gluconobacter oxydans* (GoADH). NADH regeneration is performed by Rh‐grafted carbon felt electrode with applied potential. Then the cascade enzymes PaFDH, BmFADH, PpGALS, and GoADH are well immobilized in HOF‐1 nano‐reactor. Finally, the electro‐enzymatic coupling system was successfully developed to produce EG directly from CO_2_. This electro‐enzymatic catalysis pattern offers an insight into enzyme immobilization and paves the way for complex chemical production from CO_2_ (**Scheme** **1**).

## 2 Results and Discussion

### 2.1 Cascaded Enzymatic Reaction to Produce EG from CO_2_

**Figure 1:**
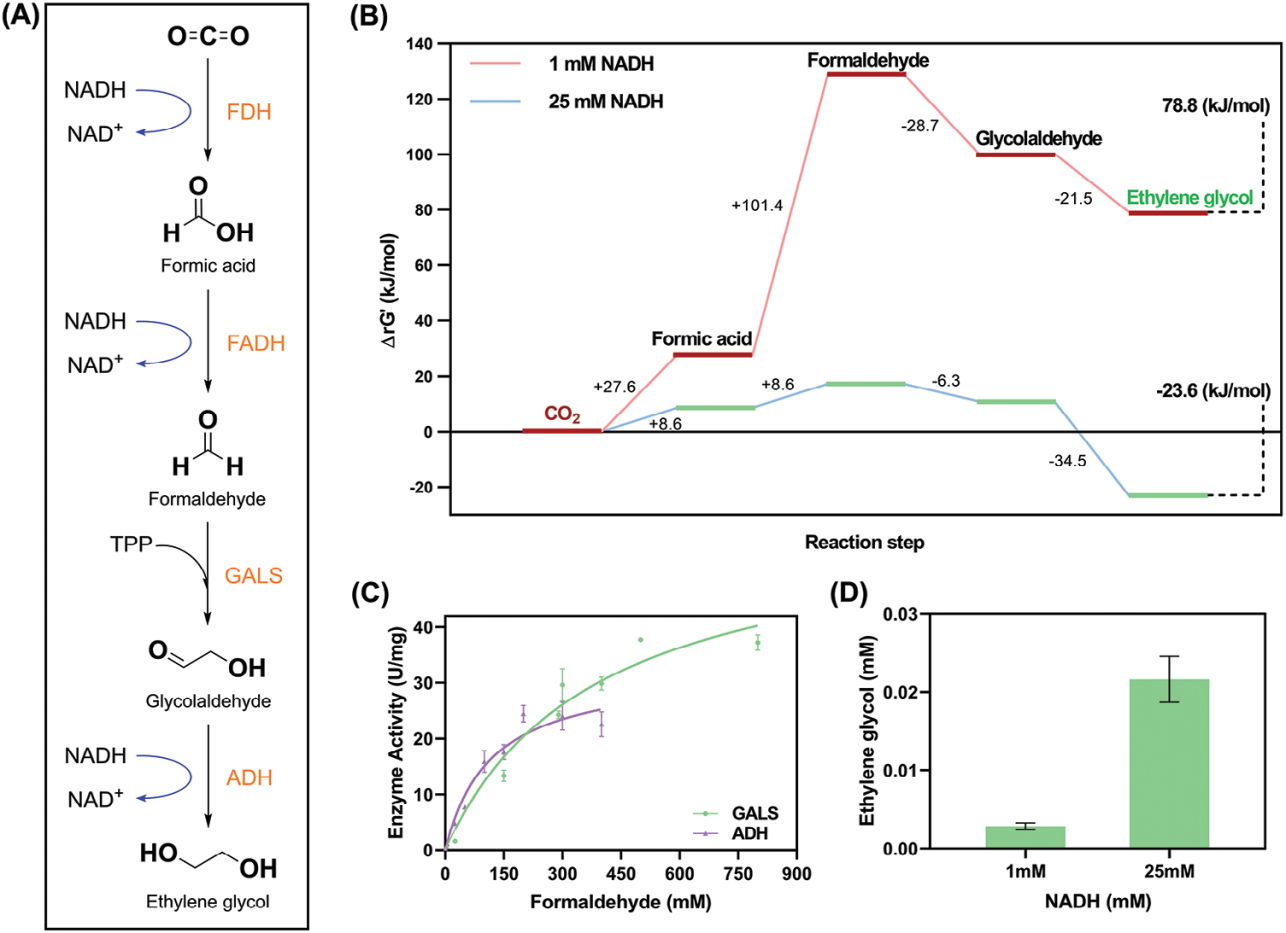
A) The new enzymatic cascade pathway for converting CO_2_ to ethylene glycol; B) Calculated Gibbs free energy change in enzymatic cascade pathway for converting CO_2_ to ethylene glycol. The red line indicates the standard Gibbs free energy (1 mM NADH) from CO_2_ to ethylene glycol is 78.8 kJ mol^−1^, while the blue line indicates the reaction Gibbs free energy change (25 mM NADH) is −23.6 kJ mol^−1^. Gibbs free energy was calculated by the website of eQuilibrator;^[^ ^24^ ^]^ C) enzyme kinetics of PpGALS and GoADH; D) the yield of ethylene glycol at different NADH concentrations.

Attempts to exploit FDH and FADH by improving their substrate affinity and catalytic efficiency have garnered rapid progress. Given that the conversion of formaldehyde into EG using GALS and ADH has been reported by a previous study,^[^ ^4^ ^]^ a multi‐enzymatic cascade system for straightforward conversion of CO_2_ to EG was thus developed through coupling two CO_2_ activation dehydrogenases with the aforementioned enzymes (**Figure** **1**A). The standard Gibbs free energy change (ΔrG′) for EG synthesis from CO_2_ in the multi‐enzymatic cascade reaction was 78.8 kJ mol^−1^, indicating that there are thermodynamic barriers to this synthetic route (Figure 1B). In particular, the initial CO_2_ activation steps had large positive ΔrG′ values of 13.8 and 50.7 kJ mol^−1^. As a result, the enzymatic CO_2_ activation to formaldehyde may be quite inefficient for the consequent reaction steps. To overcome the thermodynamic constraints associated with the uphill reaction, increasing the concentration of reduced NADH to 25 mM could reduce the cascade reaction's ΔrG′ to −23.6 kJ mol^−1^, making it thermodynamically feasible. In addition, kinetic analyses were also performed for PpGALS and GoADH in view of the competitive reactions of both enzymes for formaldehyde as substrate and intrinsic activity of GoADH toward different aldehydes, which could result in a low carbon conversion efficiency in the multi‐enzymatic cascade reaction. The GALS from *Pseudomonas putida* (PpGALS) and ADH from *Gluconobacter oxydans* (GoADH) were used to address those kinetic constraints. In the analysis of competitive reactions, PpGALS showed a 2.82‐fold higher turnover number (k_cat_ = 55.95 ± 5.946 s^−1^) than that of GoADH (k_cat_ = 19.81 ± 1.332 s^−1^), indicating that formaldehyde condensation, rather than reduction, was the dominant reaction and effectively provided driving force for the C─C formation (Figure 1C). Furthermore, upon separate addition of formaldehyde and glycolaldehyde intermediates as substrates, GoADH exhibited a higher binding affinity (K_m_) of 0.4316 mM for glycolaldehyde than that of formaldehyde, resulting in lower levels of methanol as byproduct (Figure S1, Supporting Information). What's more, GC and GC‐MS analyses of the reaction system did not reveal the presence of methanol under the existing detection conditions (Figure S2, Supporting Information). These results are consistent with the previous study that suggests ADH has a higher specificity for long‐chain aldehydes.^[^ ^25^ ^]^ Accordingly, combined results demonstrated the feasibility of EG synthesis from formaldehyde through PpGALS and GoADH acting in tandem. The minimum formaldehyde concentrations used for EG synthesis were also obtained. Therefore, EG can be synthesized even at low concentrations of 0.5 mg mL^−1^ formaldehyde, suggesting that the formaldehyde converted from enzymatic CO_2_ activation by the FDH and FADH is sufficient for the consequent reactions.

To validate the one‐pot EG synthesis from CO_2_, the CO_2_ activation was achieved by exploiting cascade dehydrogenases including FDH from *Paracoccus* sp. MKU1 (PaFDH) and FADH from *Burkholderia multivorans* (BmFADH) with high substrate affinity and catalytic efficiency in place of commercial dehydrogenases (Figure S3A, Supporting Information).^[^ ^2^, ^26^ ^]^ Meanwhile, formaldehyde condensation and further reduction used PpGALS and GoADH as mentioned (Figure S3B,C, Supporting Information). All cascade enzymes were overexpressed and purified as shown in Figure S4 (Supporting Information). With 25 mM NADH as terminal electrons and hydrogen donors, the multi‐enzymatic cascade route produced 0.016 mM EG within 6 h with an average conversion rate of 0.95 × 10^−7^ mmol CO_2_ min^−1^ mg^−1^ enzymes (Figure 1D; Figure S5, Supporting Information). This result indicated the feasibility of one‐pot EG synthesis through this multi‐enzymatic cascade system. The multi‐enzymatic cascade system contains three dehydrogenases, PaFDH, BmFADH, and GoADH, but their reduction mechanism is not identical. PaFDH compresses the CO_2_ substrate and cofactor into a conformation similar to the transition state by the favorable interaction between the amino acid, thereby completing the transfer of the proton.^[^ ^27^ ^]^ Both BmFADH and GoADH are Zn^2+^ dependent dehydrogenase enzymes that use the special tetrahedral structure formed by Zn^2+^ in the structural domains to adjust the relative positions of the substrate and cofactor to accomplish proton transfer.^[^ ^26^, ^28^ ^]^ The condensation of formaldehyde by PpGALS is similar to that of *N*‐heterocyclic carbine in chemistry, using the coenzyme thiamine pyrophosphate (TPP) to activate one aldehyde and then condense it with another^[^ ^29^ ^]^ (Figure S6, Supporting Information). To the best of our knowledge, this is the first report of the enzymatic conversion of CO_2_ to EG. Nevertheless, the low concentration of EG suggested there is still a great potential for improvement with respect to the high consumption and instability of NADH during the upgraded multi‐enzymatic CO_2_ conversion. Thus, the continuous regeneration and supply of NADH will be primarily a matter of obtaining a high EG concentration.

### 2.2 NADH Electro‐Regeneration

**Figure 2:**
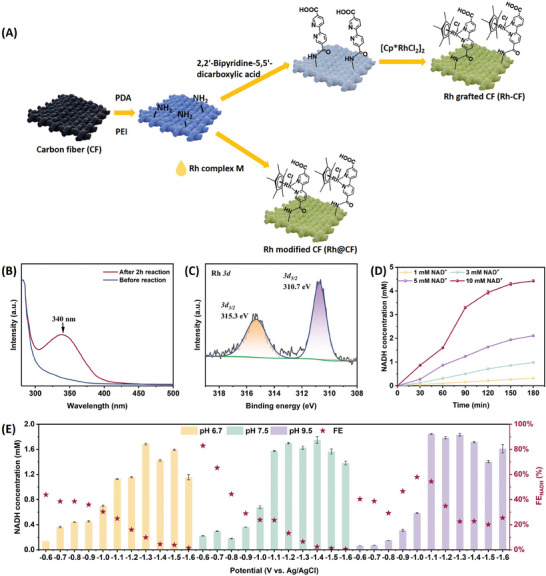
A) Schematic illustration of Rh grafted carbon felt electrode. PDA: Dopamine hydrochloride; PEI: Ethylene imine polymer; [Cp*RhCl_2_]_2_: Bis[(pentamethylcyclopentadienyl)dichloro‐rhodium. B) UV‐vis absorption spectra before and after reaction of Rh‐grafted CF (Rh‐CF). C) XPS analysis of Rh‐CF. D) NADH regeneration capacity of Rh‐CF. E) NADH regeneration under different pH and potential environments.

Among all the electrocatalysts, rhodium (Rh)‐based complexes were almost the only ideal electro‐catalyst.^[^ ^9^ ^]^ It has been identified that these Rh complexes possess proper redox potential which is slightly more negative than the redox potential of cofactor NAD^+^/NADH (i.e., −0.56 V vs SCE) but not too negative to reduce NAD^+^ into dimers (i.e., −0.9 V vs SCE).^[^ ^30^ ^]^ Besides, free Rh complexes in electrolytes may lead to both decreased Rh complexes catalytic activity and enzyme activity.^[^ ^7^, ^31^ ^]^ Immobilization of Rh complexes on electrodes could be an ideal method to separate it from enzymes partially. In this study, two immobilization methods were explored. In the first method, Rh was fixed by bipyridine on 2,2′‐bipyridine‐5,5′‐dicarboxylic acid, which was immobilized on the electrode via ‐CO‐NH‐ earlier (Rh‐CF) (**Figure** **2**A). In the second methodology, Rh complex M (M = [Cp*Rh(bpy)H_2_O]Cl, Cp = pentamethylcyclopentadienyl, bpy = 2,2′‐bipyridine‐5,5′‐dicarboxylic acid) was simply dropped onto ‐NH_2_ modified carbon electrode (Rh@CF) (Figure 2A). After 2 h electrolysis, electrolytes of Rh@CF exhibited characteristic peaks of both NADH and Rh complex (Figure S7, Supporting Information), indicating the Rh leakage from electrode.^[^ ^9^ ^]^ While only NADH peak at 340 nm can be detected in the Rh‐CF electrolyte (Figure 2B). This phenomenon indicated that catalytic active Rh complex grafted onto carbon fiber electrode via amido bond, provided tighter and stronger linkage compared to simply dropping Rh complex on carbon fiber. Rh grafted carbon fiber electrode (Rh‐CF) was then subsequently used to fulfill NADH regeneration in electro‐enzymatic EG production. The morphology of the Rh‐CF electrode was explored by SEM and the surface of carbon fiber became rough after Rh loading (Figure S8, Supporting Information). And energy‐dispersive X‐ray spectroscopy (EDS) mapping indicated that Rh complex was successfully grafted on the electrode (Figure S8, Supporting Information). The chemical composition and the valence states of the Rh complex were analyzed by X‐ray photoelectron spectroscopy (XPS) spectra (Figure S9, Supporting Information). The peaks at 310.7 and 315.3 eV were attributed to Rh 3d5/2 and Rh 3d3/2, respectively (Figure 2C),^[^ ^32^ ^]^ which was coincident with the EDS results. The performance of the prepared Rh‐CF electrode for NADH regeneration was then performed in an H‐cell before being employed in enzymatic reactions. The cyclic voltammograms (CV) of the Rh‐CF electrode with NAD^+^ were carried out to analyze the electrocatalytic activity for NAD^+^ reduction (Figure S10, Supporting Information). The bare CF electrode showed no peaks in 50 mM phosphate buffer (pH 7.50), while Rh‐CF electrode exhibited characteristic redox peaks. The reduction peak was observed at −0.82 V(vs Ag/AgCl), indicating Rh^III^ was reduced into Rh^I^.^[^ ^9^ ^]^

With the addition of NAD^+^, redox current increased significantly due to the efficient conversion of NAD^+^ to NADH.^[^ ^33^ ^]^ Upon the initial NAD^+^ concentration increasing from 0.5 to 4 mM, the cathodic current showed almost linear growth (Figure S10, Supporting Information). This tendency indicated the great catalytic capacity of Rh‐CF electrode for NADH electrochemical regeneration. The linear sweep voltammetry (LSV) curves for bare CF and Rh‐CF electrodes were then tested in CO_2_‐saturated 50 mM phosphate buffer (pH 7.5), respectively. It turned out that Rh‐CF electrode possessed a higher current density (J) and more positive onset potential than that of the bare CF electrode (Figure S10, Supporting Information). This phenomenon implied that Rh‐CF electrode has a decreased energy barrier in NADH regeneration due to Rh introduction. Furthermore, electrochemical impedance spectroscopy (EIS) was studied to investigate the electron transfer properties (Figure S10, Supporting Information). Clearly, Rh‐CF showed lower charge transfer resistance. Therefore, Rh‐grafted electrodes via amido bonds exhibited great potential in NADH regeneration.

High NADH regeneration capacity could boost the subsequent multi‐enzymatic cascade reaction. Therefore, different amounts of NAD^+^ were added to further explore the catalytic capacity of the Rh‐CF electrode (Figure 2D). The results indicated that the NADH yield increased with time and the initial concentration of NAD^+^. The Rh‐CF electrode showed excellent performance with 4.42 mM NADH regeneration after 3 h reaction (10 mM NAD^+^). The influence of applied potential and pH of electrolyte was then investigated. It was indicated that the applied potential was necessary for this NADH regeneration system (Figure S11, Supporting Information). At all pH environments, lower potential gave lower NADH concentration but higher faradic efficiency (FE) and the highest FE reached 82.9% (Figure 2E). This result suggested that higher potential was required to achieve a high NAD^+^ reduction rate. However, high potential may facilitate the side reaction, such as H_2_ evolution, leading to low FE.^[^ ^34^ ^]^ When overpotential was higher than −1.3 V (vs Ag/AgCl) in case of pH 6.7 (−1.4 V vs Ag/AgCl at pH 7.5, −1.3 V vs Ag/AgCl at pH 9.5), the NADH concentration did not increase along overpotential. This may be related to unwanted NAD· dimerization because of the lack of adsorbed H_ads_.^[^ ^35^ ^]^ Moreover, the Rh‐CF prefers to regenerate NADH in a more alkaline environment, which may be caused by the mitigation of acid‐catalyzed decay pathways for 1,4‐NADH under basic conditions.^[^ ^36^ ^]^

Overall, Rh grafted carbon fiber electrode via step‐by‐step linkage was superior to Rh deposited electrode in terms of stability. Moreover, Rh‐CF preferred pH 7.5 and pH 9.5 for NADH regeneration over pH 6.7, and −1.1 V (vs Ag/AgCl) was the optimal overpotential in consideration of both NADH yield and FE. Last but not least, Rh grafted showed higher TOF (turnover frequency) than other reported electrodes using Rh complex (Table S2, Supporting Information).

### 2.3 Enzyme Immobilization Using Hydrogen‐Bonded Organic Frameworks

**Figure 3:**
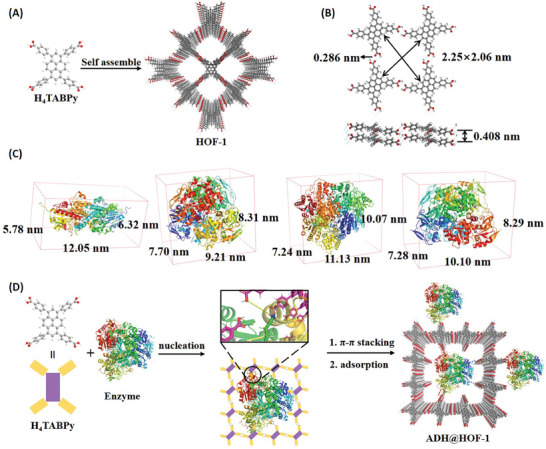
A) Schematic illustration of crystallization process of HOF‐1. B) The simulated structure and parameters of HOF‐1 in top view and side view. C) Models of PaFDH, BmFADH, PpGALS, and GoADH, respectively. D) The proposed two‐step synthesis mechanism of ADH@HOF‐1 nano‐reactor.

Efficient mass transfer is a key factor in multi‐enzymatic cascade reactions. It is supposed that multi‐enzyme immobilization by porous nano‐reactor could not only preserve its stable conformation but also facilitate mass transfer.^[^ ^8^ ^]^ However, few cases have been reported for successful co‐immobilization of multienzymes. This may be attributed to the poor cooperation between the conditions of material preparation and the characteristics of different enzymes. The proposed crystallization process of HOF‐1 and BioHOF‐1 (enzyme@HOF‐1) is illustrated in **Figure** **3**A.^[^ ^19^ ^]^ After self‐assembly, HOF‐1 was generated with 2.25 × 2.06 nm pore width and 0.408 nm layer spacing. The hydrogen bond was estimated to be 0.286 nm. Enzymes were modeled and shown in Figure 3C. It was indicated that these enzymes could not move into HOF channels directly. The proposed mechanism was shown in Figure 3D where enzyme@HOF went through 2 stages. The first one was nucleation where the carboxyl group of H_4_TABPy formed hydrogen bonds with amino acid residue on enzymes. Then H_4_TABPy molecules directionally self‐assembled into crystalline framework by strong face‐to‐face π‐π stacking.^[^ ^19^ ^]^ Some enzymes may also be adsorbed on the surface of HOF nano‐reactor through adsorption. Finally, the resulting enzyme@HOF under ambient conditions could largely retain enzyme activity when compared with COF and MOF materials.^[^ ^19^, ^21^, ^22^ ^]^ Before the cascaded enzymes (PaFDH, BmFADH, PpGALS, GoADH) for EG production from CO_2_ were attempted to be in situ immobilized in HOF‐1, GoADH was first tried for feasibility due to its convenience in large scale preparation. The rapid crystallization of H_4_TABPy in methanol and acetone solution gave bright yellow product HOF‐1 (Figure S12, Supporting Information). Moreover, a large amount of dark yellow precipitation (ADH@HOF‐1, Figure S12, Supporting Information) was formed at the moment when H_4_TABPy in DMF was added to GoADH solution. The result showed clearly that HOF‐1 exhibited the greatest enzyme capacity (990.194 mg ADH g^−1^ HOF‐1), which was about 10‐fold of the HKUST‐1, ZIF‐8, squaric acid MOF and NH_2_‐MIL MOF (Figure S13, Supporting Information). High enzyme capacity by HOF‐1 revealed its enormous potential for multienzymes immobilization by H‐bonds and π‐π stacking, compared with enzyme immobilization by adsorption (HKUST‐1, ZIF‐8, and squaric acid MOF) or by forming amido bond (NH_2_‐MIL MOF).^[^ ^37^, ^38^ ^]^

**Figure 4:**
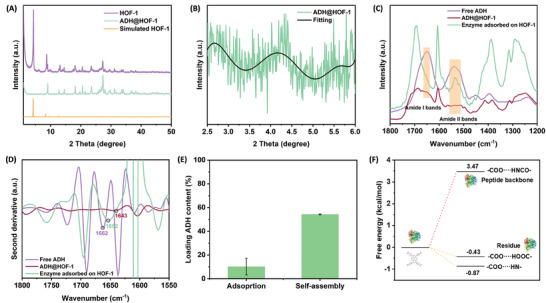
A) XRD profiles of HOF‐1, ADH@HOF‐1, and simulated HOF‐1. B) Enlarged XRD profiles of ADH@HOF‐1. C) FTIR spectra of free enzyme, ADH@HOF‐1, and enzyme adsorbed HOF‐1. D) The second derivative FTIR spectra of free enzyme, BioHOF‐1, and enzyme adsorbed HOF‐1. E) Enzyme load of HOF‐1 by adsorption and ADH@HOF by self‐assembly. F) DFT calculation of free energy between build block and different sites on protein residues.

The morphologies of HOF‐1 and ADH@HOF‐1 were explored by SEM (Figure S14, Supporting Information). Both HOF‐1 and ADH@HOF‐1 showed a stick shape with a size of around 0.5 × 6 µm. The smooth surface of ADH@HOF‐1 indicated that there was no aggregation of enzymes. The porosity was explored by low‐pressure N_2_ adsorption measurements at 77 K (Figure S15, Supporting Information). It showed typical type II isothermal with the Brunauer‐Emmett‐Teller (BET) Langmuir surface area for HOF‐1 (356.988 cm^2^ g^−1^) and ADH@HOF‐1 (185.812 cm^2^ g^−1^). These results implied that the enzyme was immobilized into the structure, which was one of the evidence for the proposed mechanism in Figure 3D. Pore size distribution showed that both HOF‐1 and ADH@HOF‐1 possessed mesoporous structure, contributing to mass transfer inside enzymes. The average pore diameter of HOF‐1 was calculated to be 2.89 nm, which was smaller than the dimension of GoADH and other enzymes (Figure 3C). This result implied that enzymes would not move into HOF pores directly. Furthermore, EDS mapping showed an obvious distribution of elements C and O which were representative of HOF‐1, yet little N and S were recognized, indicating most of the protein was inside the structure (Figure S16, Supporting Information). Confocal laser scanning microscope (CLSM) observed that the RhBTC (Rhodamine B) labeled enzymes showed red fluorescence in a dark field (Figure S17, Supporting Information). These images indicated that enzymes were successfully immobilized. The phase purity was confirmed by powder X‐ray diffraction (PXRD, **Figure** **4**A,B). It showed that HOF‐1 structure was consistent with the simulation results and after enzyme incorporation the peak at 4.3 degrees decreased largely, indicating declined crystallinity in <0 1 1> direction. The results showed that the protein secondary structure was well maintained. After encapsulation, the thermogravimetry analysis (TGA) was conducted (Figure S18, Supporting Information). ADH@HOF‐1 started to lose weight at about150 °C while HOF‐1 maintained until 400 °C. This difference could be attributed to protein decomposition at high temperatures. All the characterizations implied that ADH was successfully immobilized into HOF‐1. To further confirm the proposed mechanism, FTIR experiments were conducted to detect protein structure change. As shown in Figure 4C, ADH@HOF‐1 showed spectral signals C═O (1650 cm^−1^), the bending vibration of N─H (1550–1530 cm^−1^), and the stretching vibration of C─N (1400 cm^−1^), Which could be attributed to preserved typical amide I band and amide II band after protein was embedded inside, indicating successful immobilization by a protein‐induced self‐assembly process.^[^ ^19^ ^]^ Moreover, the obtained HOF‐1 after enzyme adsorption showed amide bands. As discussed above, the dimensions of enzymes and HOF‐1 pores were not matched, so it was speculated that enzymes could be immobilized with amino acids on the surface by forming hydrogen bonds. The second‐order spectrum obtained after Fourier deconvolution is shown in Figure 4D. Obviously, the characteristic peak at 1662 cm^−1^ in free enzyme was red shifted ca. 10 cm^−1^ in enzyme‐adsorbed HOF‐1 and ca. 19 cm^−1^ in ADH@HOF‐1. This result implied that there was hydrogen bond interaction between enzymes and surface amino acids. Therefore, the enzyme would form hydrogen bonds with HOF molecules, and some would be embedded into HOF structure via π‐π stacking as described above, while others would be immobilized on surface. Except for forming hydrogen bonds, adsorption which was the instinct of porous material may also contribute to enzyme immobilization. Furthermore, enzyme adsorption experiments were conducted and it turned out there were about10% free enzymes adsorbed on HOF‐1, even though the loading amount was much smaller than ADH@HOF‐1 (Figure 4E). Based on analysis before, these enzymes would not move into HOF pores directly, yet there were 10% of enzymes were immobilized. This led to a hypothesis that a small amount of enzyme would be fixed onto the HOF surface area as indicated in Figure 3D.

Density functional theory (DFT) calculation was carried out to further investigate the active site for hydrogen bonding (Figure 4F). Glycine, lysine, and glutamic acid were chosen as model amino acids for amide bond (N─H) at peptide backbone, ‐NH at residue, and ‐COOH residue to form hydrogen bond with ‐COOH on H_4_TABPy molecule. The free energy changes (ΔrG′) were calculated to be 3.47, −0.87 and −0.43 kcal mol^−1^, respectively (in phosphate buffer, pH 7.5), suggesting that H_4_TABPy would form hydrogen bonds with ‐NH and ‐COOH on amino acid residues rather than amide bond in the peptide backbone. It was worth noting that water environment seems to destroy HOF structure because ‐COOH in H_4_TABPy was deprotonated and the intramolecular hydrogen bond interaction of H_4_TBAPy is quite weak.^[^ ^19^ ^]^

Optimal reaction pH was then explored (Figure S19, Supporting Information). It showed that free ADH had the best activity at pH 9.5 while ADH@HOF‐1 preferred pH 6.7. The organic solvent resistance, storage stability, and circulation stability were also tested (Figure S19, Supporting Information). ADH@HOF showed great resistance toward organic solvents (acetone, ether, dichloromethane, ethyl acetate, tetrahydrofuran). Moreover, ADH@HOF showed excellent activity after 6 days as well as 5 cycles. In conclusion, HOF scaffold could not only retain the activity but also prevent enzymes from organic solvents.

### 2.4 Electro‐Driven Cascaded Enzymatic Conversion of CO_2_ to EG in Nano‐Reactor

**Figure 5:**
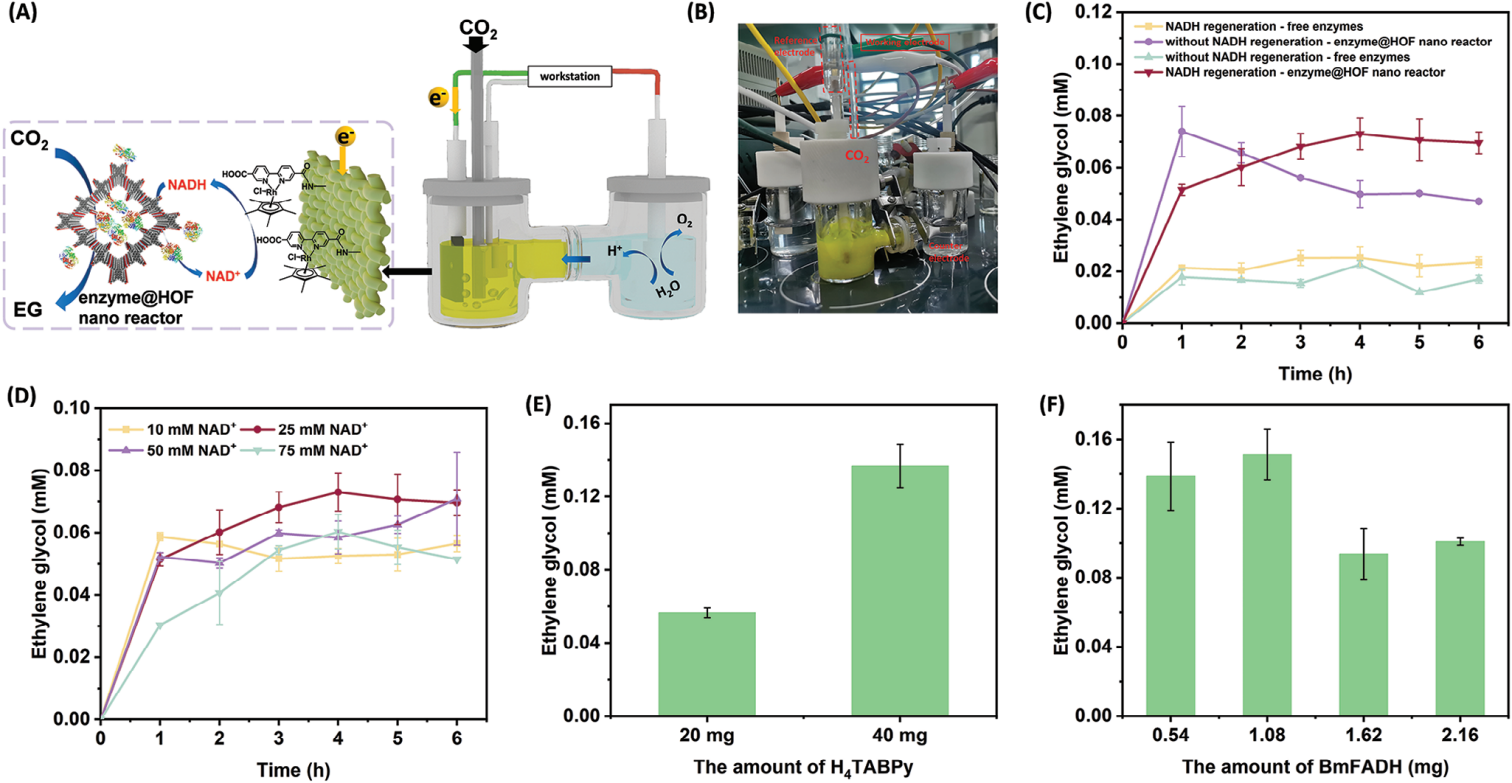
A) Schematic of enzyme‐electro hybrid catalysis system. B) Digital photo of enzyme‐electro hybrid system. C) Ethylene glycol production from CO_2_ with and without NADH regeneration. For NADH regeneration, 25 mM NAD^+^, 5 mM TPP, and 5 mM MgCl_2_ were added, for comparison, 25 mM NADH, 5 mM TPP, and 5 mM MgCl_2_ were added and reaction was conducted without NADH regeneration. D) Influence of initial NAD^+^ concentration on EG yield. E) Increasing HOF material leading to exponential EG yield. F) Optimization of the BmFADH amount to EG yield.

Before integrating EG enzymatic pathway with NADH regeneration, enzymatic conversion of CO_2_ to formate with FDH and methanol with FDH, FADH, and ADH were tried First for feasibility in the developed electro‐enzyme coupling system, respectively (Figures S20 and S21, Supporting Information). The result showed that this system can successfully produce formate and methanol from CO_2_. Especially, the methanol yield could reach up to 0.25 mM after 6 h reaction, which is the highest currently reported.^[^ ^33^ ^]^ Finally, multi‐enzymatic conversion of CO_2_ to EG in the HOF nano‐reactor was then integrated with electro‐NADH regeneration, as shown in **Figure** **5**A,B. As shown in Figure 5C, EG yield would decrease over time when there is no electro‐based regeneration, indicating that without external interference, the reversible reaction would follow the path where EG was consumed but not accumulated. While with NADH regeneration, EG yield was accumulated slowly at first few hours, indicating that electro‐driven NADH regeneration would drive the reaction to go along the direction where EG was accumulated. With time moving on, EG yield became slow and sometimes even decreased, implying that the rate of EG production and consumption reached balance. Long time stability had also been investigated (Figure S22, Supporting Information). It turned out that EG would accumulate under continuous operation conditions within 48 h.

To increase EG yield, we have tried some possibilities, as shown in Figure 5D–F. The concentration of NAD^+^ was first explored since NADH concentration had a great impact on Gibbs free energy. As shown in Figure 5D, 25 mM NAD^+^ reached the highest EG yield, indicating that a proper amount of NADH would benefit EG production while high concentration would hinder it. Then, 3 times the amount of initial enzymes were added to increase EG yield. However, it turned out that there was no significant increase in the yield, as in Figure S23 (Supporting Information). This indicated that the amount of enzyme would not be the constraint. Interestingly, when immobilizing enzymes in a more dispersive way (Figure S24, Supporting Information), the EG yield was increased obviously (Figure 5E). The dispersed immobilization of the cascade enzyme is able to consume more NADH by improving mass transfer, which in turn increases the concentration of generated product.

As discussed in Figure 1, formate to formaldehyde catalyzed by FADH was the most thermal‐dynamic unfavorable step, thus the optional amount of FADH was explored. It turned out that high FADH concentrations would benefit formaldehyde to formate and the proper FADH addition was 1.08 mg and EG yield could reach 0.15 mM EG until 6 h (Figure 5F). The average conversion rate was calculated to be 7.15 × 10^−7^ mmol CO_2_ min^−1^ mg^−1^ enzyme, which was far beyond the CO_2_ conversion rate without electro NADH regeneration in Figure 1D (0.95 × 10^−7^ mmol CO_2_ min^−1^ mg^−1^ enzyme), indicating higher enzymatic catalyst efficiency in overall process. This could be attributed to high NADH concentration generated by electrocatalysis lowering the Gibbs free energy. The recycle stability of Rh‐CF and enzyme@HOF nanoreactor was also investigated (Figures S25 and S26, Supporting Information). It turned out that Rh‐CF electrode remained more than 60% activity after 6 cycles. It indicated that Rh was bonded on the electrode tightly. The enzyme@HOF nanoreactor exhibited 40% activity after 6 cycles. The decrease may be caused by dissociation of HOF and leakage of enzymes while being centrifuged.

This study is the first attempt to obtain EG in a one‐pot multi‐enzymatic cascade process that is directly from CO_2_ gas molecules with a combination of electrocatalytic NADH regeneration. This catalytic system might be instructive for the production of other value‐added chemicals from CO_2_. Low solubility of CO_2_ in aqueous solution has been the concern and efforts have been made to convert it into methanol for convenience in enzymatic reaction.^[^ ^39^ ^]^ It's reasonable to believe that the yield of EG will be greatly increased if the concentration of formate or methanol is increased. In addition, the complexity of the multi‐enzyme cascade reaction accounts for the very slow synthesis. Future work will concentrate on improving enzyme activity and tolerance through genetic engineering, synthetic biology, etc., as well as designing more efficient CO_2_ reduction and NADH regeneration electrodes, to have a positive effect on the system efficiency.

## 3 Conclusion

In this study, a new metabolic pathway from CO_2_ to EG via multi‐enzymatic cascade with PaFDH, BmFADH, PpGALS, and GoADH has been developed. This route could produce 0.016 mM EG within 6 h at an average conversion rate of 0.95 × 10^−7^ mmol CO_2_ min^−1^ mg^−1^ enzyme, using 25 mM NADH as terminal electrons and hydrogen donors. Then, Rh functionalized electrode with high FE (82.9% at −0.6 V vs Ag/AgCl) was constructed for NADH regeneration and cyclic utilization continuously by providing the proton and electron for this multi‐enzymatic cascade reaction. In order to further shorten the electron transfer route of the multi‐enzymatic cascade reaction, four enzymes were first co‐immobilized into HOF‐1 nano‐reactor by crystallizing via self‐assembly in the ambient water environment. Finally, 0.15 mM EG (average conversion rate: 7.15 × 10^−7^ mmol CO_2_ min^−1^ mg^−1^ enzyme) was achieved from CO_2_ in such electro‐driven multi‐enzymatic cascade nanoreactor in 6 h. Therefore, this study paves a new way for upcycling CO_2_ into value‐added products and enlightens electro‐biocatalysis hybrid systems in the future.

## 4 Experimental Section

### Materials

Carbon fibers were purchased from Suzhou Sinero Technology Co., Ltd. Dopamine hydrochloride (PDA, CAS: 62‐31‐7) was purchased from Sigma‐Aldrich. Ethylene imine polymer (PEI, CAS: 9002‐98‐6) was purchased from MACKLIN. N‐(3‐Dimethylaminopropyl)‐N′‐ethylcarbodiimide hydrochloride (EDC, CAS: 25952‐53‐8) and N‐Hydroxysuccinimide (NHS, CAS: 6066‐82‐6) were purchased from Aladdin. Bis[(pentamethylcyclopentadienyl)dichloro‐rhodium ([Cp*RhCl_2_]_2_, CAS: 12354‐85‐7), 2,2′‐bipyridine‐5,5′‐dicarboxylic acid (CAS: 1802‐30‐8), acetamide (CAS: 60‐35‐5) and 1,3,6,8‐Tetra(4′‐carboxyphenyl)pyrene (H4TBAPy, CAS: 933047‐52‐0) were purchased from Adamas‐beta. Tris HCl (CAS: 77‐86‐1), thiamine pyrophosphate (CAS: 154‐87‐0), and 2‐(N‐Morpholino) ethanesulfonic acid monohydrate (MES, CAS: 145224‐94‐8) were purchased from Solarbio. Dichloromethane (CAS: 75‐09‐2) was purchased from General‐reagent. Ethanol and methanol were purchased from Aladdin. Acetone (CAS: 67‐64‐1) was purchased from Xilong Scientific Co., Ltd. Diethyl ether anhydrous (CAS: 60‐29‐7), and acetic anhydride (CAS: 108‐24‐7) was purchased from Sinopharm Chemical Reagent Co., Ltd. Nafion‐117 (CAS: 31175‐20‐9) was purchased from Sigma‐Aldrich. N, N‐Dimethylformamide (DMF, CAS: 68‐12‐2), and Rhodamine B (RhHB, CAS: 36877‐69‐7) were purchased from MACKLIN. Coomassie Plus Protein Assay Reagent (Product number: 23 236) was purchased from Thermo Scientific. Citric acid (CAS: 77‐92‐9) and 2‐Propanol (CAS: 67‐63‐0) were purchased from Adamas‐beta. Sodium formate (CAS: 141‐53‐7), NAD^+^ (CAS: 20111‐18‐6) and NADH (CAS: 606‐68‐8), glycolaldehyde dimer (CAS: 23147‐58‐2), ethylene glycol (CAS: 107‐21‐1) and magnesium chloride (CAS: 7786‐30‐3) were purchased from Aladdin. Formaldehyde solution (16%, w/v, methanol free, CAS: 50‐00‐0) was purchased from Thermo Scientific. All the chemicals were used without further purification.

### Characterization

The morphology of the samples was examined using a Hitachi S‐4800 field emission scanning electron microscope (SEM) at 5 kV and 10 µA and Elemental mapping was carried out by energy dispersive X‐ray spectroscopy (EDS) (Inca X‐MAX, Oxford, UK). Crystallinity and crystal structure of some samples were characterized by powder X‐ray diffraction (XRD) (Smartlab, Japan). The ultraviolet‐visible (UV‐vis) absorbance measurements were detected by Agilent BioTek Cytation 5 detector at 340 nm. The Fourier transform infrared (FTIR) spectra were measured with an FTIR spectrometer (Nicolet 380, Thermo Scientific). X‐ray photoelectron spectroscopy (XPS) was recorded using an Escalab 250Xi instrument (Thermo Scientific) equipped with an Al Kα microfocused X‐ray source and the C1s peak at 284.6 eV as the internal standard. Thermogravimetric analyses (TGA) were performed under N_2_ atmosphere (20 mL min^−1^) with temperature increasing at 10 °C min^−1^ using a TA‐Q50 system. Circular dichroism (CD) spectra were analyzed by a J1700 CD Spectrometer (JASCO, Japan) in the spectrum region 190–300 nm. Confocal laser scanning microscope (CLSM) was conducted by Leica TCS SP 5 (Germany). The specific surface area of the membrane was generally detected by nitrogen adsorption and desorption instruments (ASAP 2020 PlusHD88, America). Electrochemical characterization was carried out by CHI660E workstation using a three‐electrode system in an H‐cell.

### Heterologous Expression, Purification, and Protein Quantification of PaFDH, BmFADH, PpGALS, and GoADH

Constructing plasmids pETDuet‐PaFDH, pETDuet‐BmFADH, pETDuet‐PpGALS, pETDuet‐GoADH by inserting genes with C‐terminal Histag into the NcoI/SacI restriction sites of the pETDuet plasmids respectively. And transferred the constructed plasmids into *E. coli* BL21(DE3). The above four genes were codon‐optimized and synthesized by Shanghai Generay Biotechnology company. Protein purification using the method described by *Huang* et al.^[^ ^40^ ^]^ The protein concentration was determined by Pierce BCA Protein Assay Kit (23 225, Thermo Scientific) and BSA was used as standard, and the protein purity was determined by SDS‐PAGE.

### Activity assay of PaFDH, BmFADH, PpGALS, and GoADH

The activity of PaFDH, BmFADH, and GoADH was determined by measuring the consumed NADH in the reaction system with BioTek Cytation 5 imaging reader at 340 nm. In PaFDH reaction system, 0.20 mg mL^−1^ PaFDH and 1.0 mM NADH were added into PB buffer (50 mM, pH 7.5), and CO_2_ was bubbled straightly as the substrate for 1 h. The BmFADH reaction system contains 0.20 mg mL^−1^ BmFADH, 1 mM NADH, and 5 mM NaMeO_2,_ and the reaction was carried out in PB buffer (50 mM, pH 7.5) for 2 h. Similarly, the activity of GoADH was tested in PB buffer for 1 h, and the reaction system contained 0.2 mg mL^−1^ GoADH, 1 mM NADH, and 50 mM glycolaldehyde.

The activity of PpGALS was determined by measuring the yield of glycolaldehyde. The reaction was carried out in PB buffer (50 mM, pH 7.5) and contained 0.20 mg mL^−1^ PpGALS, 1 mM TPP, 5 mM MgCl_2,_ and 50 mM formaldehyde. After reacting for 1 h, the reaction mixture was detected by gas chromatograph (GC‐2030, Shimadzu, Japan) with a BID detector and a SH‐WAX column (60 m × 0.32 mm × 1.0 µm). Using an external standard method, the glycolaldehyde concentration was calculated from the area corresponding to the characteristic peak of glycolaldehyde observed in the chromatogram.

### Kinetic analysis of PpGALS and GoADH

The initial continuous assay system of PpGALS was the same as its activity assay but with different concentrations of formaldehyde (25 to 800 mm). The assay system of GoADH contains 1 mm NADH, 0.2 mg mL^−1^ GoADH, and different concentrations of formaldehyde (5 to 400 mm). After reacting for 10 min at 25 °C, the residual formaldehyde in the reaction mixture was detected at 420 nm after reacting with Nash reagent at 58 °C, 5 min. Nash regent was prepared by dissolving 5 m ammonium acetate and 50 mM acetylacetone.^[^ ^41^ ^]^ One unit of formaldehyde conversion activity was defined as the PpGALS and GoADH amount required to consume 1 µmol of formaldehyde per minute under reaction conditions. *K_m_* was curve‐fitted according to the Michaelis‐Menten equation, which is determined by GraphPad Prism 8. All experiments were conducted in triplicate.

### Synthesizing Ethylene Glycol from CO_2_ Through Enzymatic Cascade Pathway

The synthesis of ethylene glycol from CO_2_ was carried out in PB buffer (50 mM, pH 7.5, 25 °C). The reaction system contains 0.4 mg mL^−1^ PaFDH, 0.8 mg mL^−1^ BmFADH, 0.8 mg mL^−1^ PpGALS, 0.2 mg mL^−1^ GoADH, 25 mM NADH, 1 mM TPP, and 5 mM MgCl_2_. CO_2_ was bubbled straight into the reaction system as substrate.

### Synthesis of Rh Grafted Carbon Fiber Electrode (Rh‐CF)

Carbon fibers were cut into 1 × 2 cm^2^ pieces and then sonicated with soap water, ethanol, and acetone for 15 min, respectively. The carbon fibers were then put into a mixture of PDA and PEI to introduce ‐NH_2_ on the surface.^[^ ^42^ ^]^ Typically, 40 mg PDA and 40 mg PEI were mixed and dissolved in 20 mL Tris HCl (50 mM, pH 8.5) under stirring, then carbon fibers were put into the mixture for 6 h. Simultaneously, 78 mg EDC, 78 mg NHS, and 98 mg 2,2′‐bipyridine‐5,5′‐dicarboxylic acid were dissolved in 20 mL MES (50 mM, pH 5.0) solution and stirred for 1 h. After 6 h pretreatment, carbon fibers were washed with deionized water and then put into MES solution for another 90 min. After washed with deionized water, carbon fibers were then put into the solution containing 20 mg [Cp*RhCl_2_]_2_ (Cp = pentamethylcyclopentadienyl) and 20 mL dichloromethane. After stirring for 3 h, carbon fibers were washed with deionized water and then went through vacuum drying for 12 h at 60 °C.

### Synthesis of Rh Complex M and Rh Dropped Carbon Fiber Electrode (Rh@CF)

The synthesis of Rh complex **M** was based on literature^[^ ^43^ ^]^ with slight modification. First, 61.8 mg [Cp*RhCl_2_]_2_ was suspended in 1 mL of anhydrous methanol, and 48.84 mg 2,2′‐bipyridine‐5,5‐dicarboxylic acid was added. The suspension then became clear, forming a pale yellow solution. Diethyl ether anhydrous was added dropwise to the solution at 4 °C until the precipitation of orange‐yellow particles appeared. Finally, the obtained precipitate was dried under vacuum at 80 °C for 2 h.

For Rh@CF, Rh complex **M** (1 mg) was mixed with 100 µL methanol and sonicated for 20 min. Then the solution was drop‐added onto PDA and PEI‐treated carbon fiber. After drying under vacuum for 12 h at 60 °C, Nafion‐117 was dropped onto electrode. Finally, Rh@CF was dried at ambient environment.

### Synthesis of HOF‐1, ADH@HOF‐1, and BioHOF‐1

HOF‐1 was synthesized according to the literature with some modifications.^[^ ^19^ ^]^ 200 mg H_4_TBAPy was dissolved in 60 mL DMF and stirred for 2 h at 120 °C until the yellow solution became clear. After cooling down to room temperature, 160 mL acetone was added to the solution and the mixture was stirred at ambient environment for 12 h. The suspension was filtered and washed with methanol and then dried under vacuum at 60 °C for 12 h.

ADH@HOF‐1 and BioHOF‐1 were also synthesized according to the literature with some modifications.^[^ ^19^, ^44^ ^]^ 20 mg H_4_TBAPy was dissolved into 2 mL DMF and sonicated for 20 min until the mixture became clear. For ADH@HOF‐1, 0.5 mL of as prepared ADH solution (10.56 mg mL^−1^) and 1 mL phosphates buffer (50 mM, Ph 7.5) were added into 0.4 mL H_4_TBAPy solution. The mixture was then put into 4 °C refrigerator and aged for 20 min. The precipitates were then collected by centrifugation at 12 000 rpm and washed by methanol 3 times.

For multi‐enzyme immobilization (namely, BioHOF‐1), enzyme solution (200 µL FDH, 8.418 mg mL^−1^; 200 µL FADH, 2.7 mg mL^−1^; 100 µL GALS, 13.825 mg mL^−1^; 100 µL ADH, 10.56 mg mL^−1^) was mixed with 1 mL H_4_TBAPy solution in 1.6 mL centrifuge tube. The other steps were in the same with ADH@HOF‐1. The precipitates of 2 centrifuge tubes were added for reaction.

### Enzyme Loading Calculation

The protein concentration was examined by Bradford method.^[^ ^45^ ^]^ Typically, 10 µL sample was added to 96‐wall plate, and 300 µL Coomassie Plus Protein Assay Reagent was then added. The mixture was incubated at room temperature for 10 min and then detected by UV‐Vis spectrophotometer. The concentration of protein was directly proportional to the signal at 595 nm.

### Electro‐Catalysis NADH Regeneration

Electrochemical NADH regeneration was carried out by CHI660E workstation using a three‐electrode system in an H‐cell. Rh‐modified carbon fiber, Pt wire, and Ag/AgCl/KCl (3 m) were used as working electrodes, counter electrodes, and reference electrodes, respectively. The scan rate was 5 mV s^−1^. Anodic chamber and cathodic chamber were filled with 10 mL phosphates buffer (50 mM, pH 6.7 and pH 7.5) and Tris HCl (50 mM, pH 9.5) as an electrolyte which was degassed by N_2_ before use. The working electrode was set into constant potential and i–t curve was recorded during reaction. Different amounts of NAD^+^ were added as substrate. NADH was detected by Agilent BioTek Cytation 5 detector at 340 nm. The amount of electrochemical active Rh site (N) was estimated by integration of the charge (Q) using CV curves.

(1) N=Q/2F

where F is the Faraday constant. The turnover frequency (TOF) was calculated by

(2) TOF=I/2NF

where I is catalytic current, F is the Faraday constant.

### Enzymatic Electro‐Catalysis for Formate, Methanol, and Ethylene Glycol Production From CO_2_

Enzymatic electrochemical reaction was conducted in conventional three electrodes H cell using CHI660E workstation. Rh grafted carbon fiber, Pt wire, and Ag/AgCl/KCl (3 m) was used as working electrode, counter electrode, and reference electrode, respectively. Anodic chamber and cathodic chamber were filled with 10 mL phosphates buffer (50 mM, pH 7.5) as an electrolyte which was degassed by CO_2_ before use. For format production, FDH was immobilized using the method in ADH@HOF‐1, replacing ADH with FDH, and then transferred into cathodic chamber. NAD^+^ (1 mM) was added into electrolyte as substrate. CO_2_ was bubbled into electrolyte during reaction. Constant potential was applied onto working electrode and i–t curve was collected. For methanol production, cascaded enzymes (FDH, FADH, and ADH) were immobilized using the mentioned method above, as well. The other reaction conditions were consistent with formate production. For EG production, BioHOF‐1 was transferred into a cathodic chamber. Except for NAD^+^, MgCl_2_ (500 µL, 500 m) and TPP (200 µL, 250 mM) were also added. The other reaction conditions were consistent with that mentioned above unless otherwise noted.

### Enzyme Adsorption by HOF‐1 Experiment

HOF‐1 (4 mg) was added into 1.9 mL phosphates buffer and sonicated for 10 min, then 0.4 mL ADH solution (10.56 mg mL^−1^) was added. After aging in 4 °C refrigerator for 20 min, the mixture was centrifugation at 12 000 rpm and washed by methanol 3 times, the supernatant was collected. The free protein concentration was quantified by Bradford method. And the amount of fixed protein was determined by the difference of the initial concentration and free protein concentration.

### Fluorescence Labeling of Proteins

Briefly, ADH solution (5 mL, 1 mg mL^−1^) was mixed with 1 mg Rhodamine B (RhHB). The mixture was then stirred in dark conditions for 12 h. Finally, the RHB‐labeled enzyme was obtained by ultrafiltration 3 times with a centrifugal filtration device (molecular weight cut‐off MWCO = 8 kDa) to remove the excess fluorescent impurities.

### Production Analysis

The concentration of formic acid was measured by UV spectrophotometer. Sodium formate dissolved in phosphate buffers (100 mM. pH 7.0) was used for standard curve establishment. Solution A was prepared by dissolving 0.5 g citric acid and 10 g acetamide in 100 mL isopropyl alcohol, and solution B was prepared by dissolving 30 g sodium acetate in 100 mL water. The sample containing formate (100 µL) was then mixed with 0.2 mL solution A, 10 µL solution B, and 0.7 mL 100% acetic anhydride and incubated at 50 °C for 2 h. Yellow solution was produced and photometric measurements were made at 515 nm. The standard curve is shown in Figure S30 (Supporting Information).

Methanol and EG were detected by a SHIMADZU GC‐2030 equipped with an SH‐Wax column and BID detector.

### DFT Calculation

To assess the binding free energies for amino acid residue (A) and organic linker (B), glycine, lysine, and glutamic acid were chosen as model amino acids for amide bond (N─H) at peptide backbone, ‐NH at residue and ‐COOH residue respectively to form hydrogen bond with ‐COOH on H_4_TABPy and the binding free energies were calculated according to:

(3) $$\Delta G\left(\mathrm{AB}\right)=G{\left(\mathrm{AB}\right)}_{\mathrm{aq}}-\left(G{\left(A\right)}_{\mathrm{aq}}+G{\left(B\right)}_{\mathrm{aq}}\right)$$

G(AB)_aq_, G(A)_aq,_ and G(B)_aq_ were calculated from the frequency calculations in water. All electronic structure calculations were done in Gaussian 16 revision A.03.

The structure of HOF‐1 was simulated by Material Studio.

### The average conversion rate of CO_2_

The average conversion rate of CO_2_ was calculated by:

(4) $$X\;=\;(2\;•\;M_{\mathrm{EG}}\;•\;V)/(T\;•\;m)$$

Where M_EG_ was EG yield, V was volume of electrolyte, T was reaction time, and m was enzyme mass.

## Conflict of Interest

The authors declare no conflict of interest.

## Author Contributions

L.L. and Y.Z. contributed equally to this work. The authors designed the experiments and wrote the original draft; Y.H. and S.Z. conceived the idea, provided financial support, and supervised the project; Y.H. and X.J. helped polish the draft and discussed the experiment; S.S. helped with HOF synthesis; B.G. helped with the docking simulation.

## Supporting information

Supporting Information

## Data Availability

The data that support the findings of this study are available from the corresponding author upon reasonable request.
