# Supplementary material for: Electro‐Driven Multi‐Enzymatic Cascade Conversion of CO2 to Ethylene Glycol in Nano‐Reactor
Source: Adv Sci (Weinh). 2024 Sep 4;11(41):2407204. doi: 10.1002/advs.202407204 (PMC11538636; doi:10.1002/advs.202407204)
Supplement: Supplementary file 1 — Supporting Information [file ADVS-11-2407204-s001.docx]

Supporting Information

Electro-Driven Multi-Enzymatic Cascade Conversion of CO_2_ to Ethylene Glycol in Nano-Reactor

*Likun Luan†, Yingfang Zhang†, Xiuling Ji, Boxia Guo, Yuhong Huang* , Suojiang Zhang**


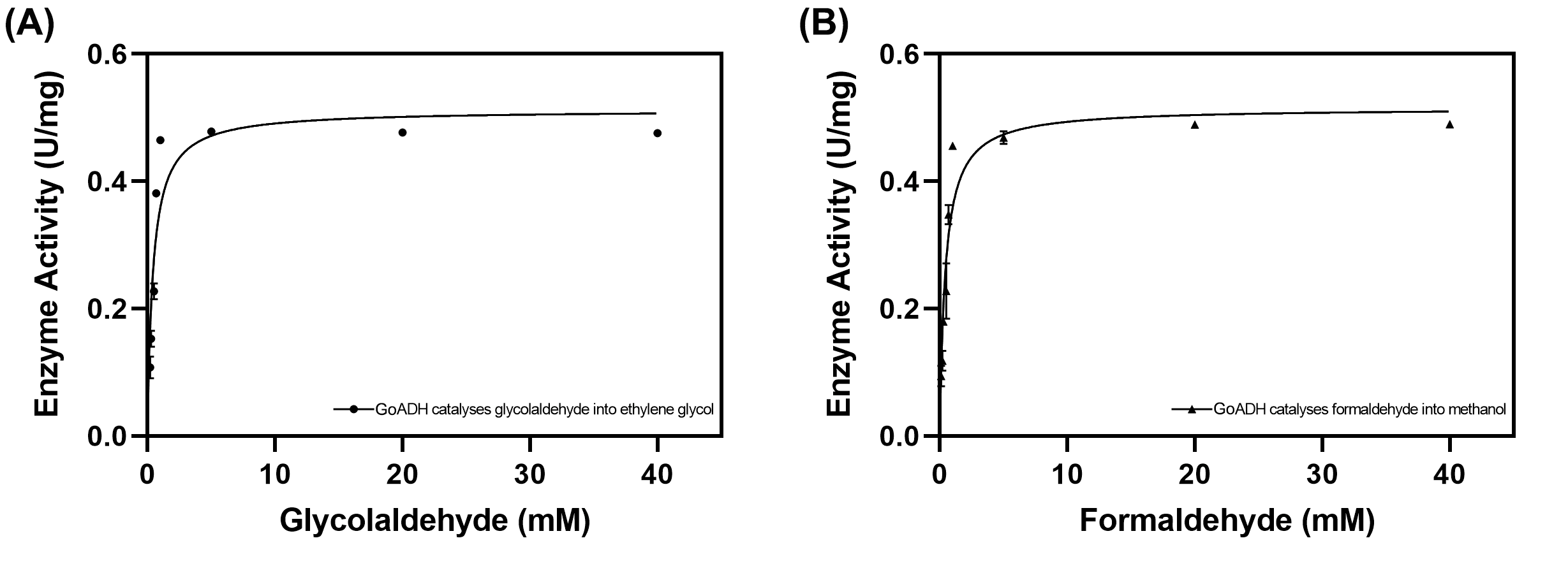


**Figure S1.** Enzyme kinetics of GoADH. (A) Enzyme kinetics of GoADH catalyzing glycolaldehyde into ethylene glycol (K_m_=0.4316 ± 0.07276 mM). The initial continuous assay system contains 1 mM NADH, 0.2 mg/mL GoADH and different concentrations of glycolaldehyde (0.2 to 40 mM). After reacting in PB buffer (50 mM, pH 7.5, 25 °C) for 10 minutes, the residual NADH in reaction mixtures were detected at 340 nm. (B) Enzyme kinetics of GoADH catalyzing formaldehyde into methanol (K_m_=0.4416 ± 0.05491mM). The initial continuous assay system contains 1 mM NADH, 0.2 mg/mL GoADH and different concentrations of formaldehyde (0.1 to 40 mM). After reacting in PB buffer (50 mM, pH 7.5, 25 °C) for 10 minutes, the residual NADH in reaction mixtures were detected at 340 nm. One unit of glycolaldehyde and formaldehyde conversion activity were defined as the GoADH amount required to consume 1 μmol of NADH per minute under reaction conditions. *Km* were curve-fitted according to the Michaelis-Menten equation, which is determined by GraphPad Prism 8. All experiments were conducted in triplicate.


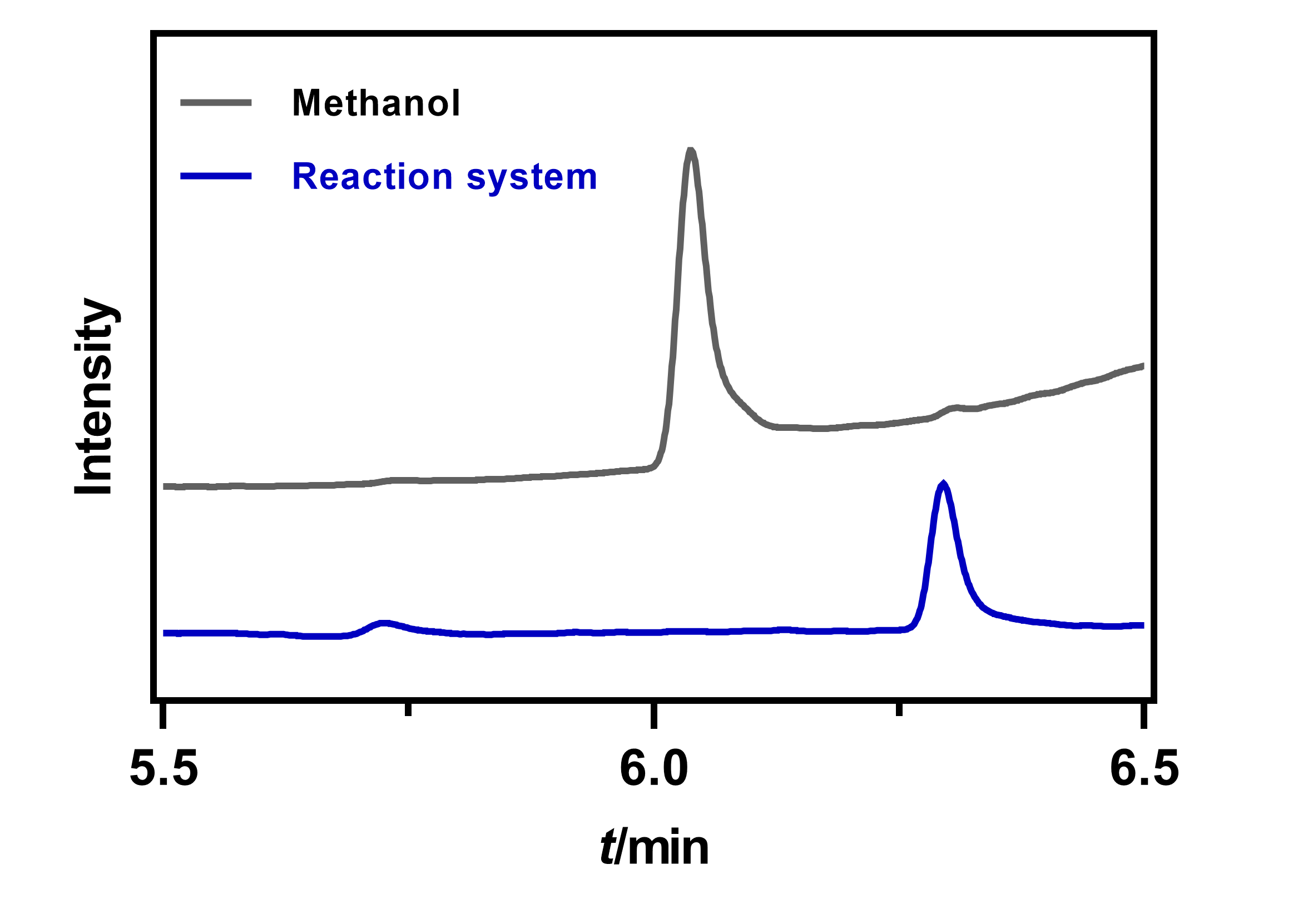


**Figure S2.** GC spectrum of methanol and the reaction system solution of CO_2_ to ethylene glycol.


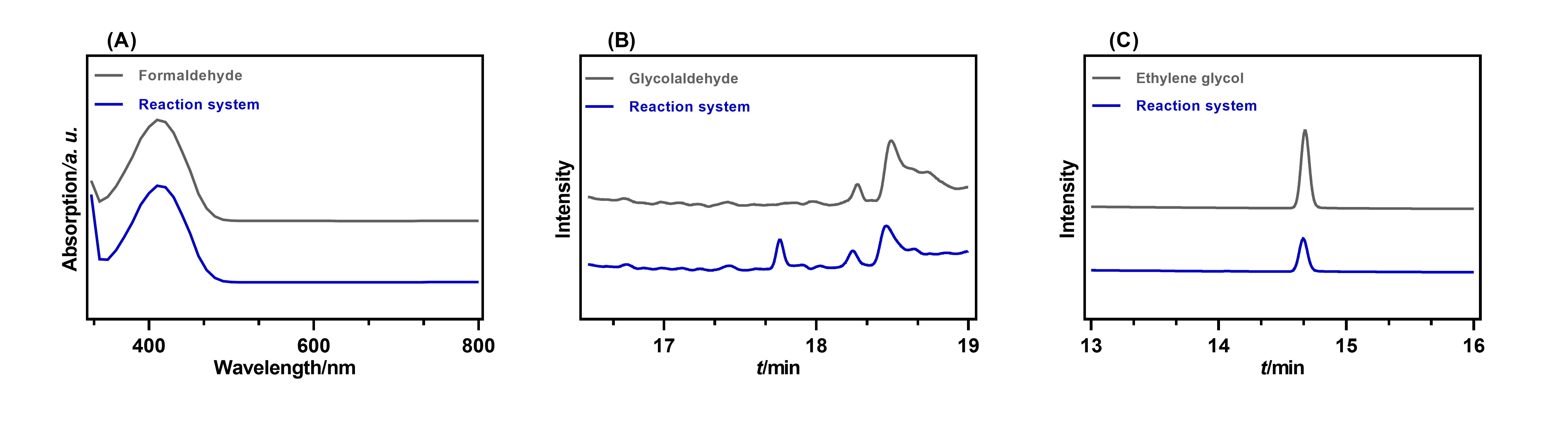


**Figure S3.** (A) Absorption spectrum of formaldehyde and the reaction system solution of CO_2_ to formaldehyde. (B) GC spectrum of glycolaldehyde and the reaction system solution of formaldehyde to glycolaldehyde. (C) GC spectrum of ethylene glycol and the reaction system solution of glycolaldehyde to ethylene glycol.


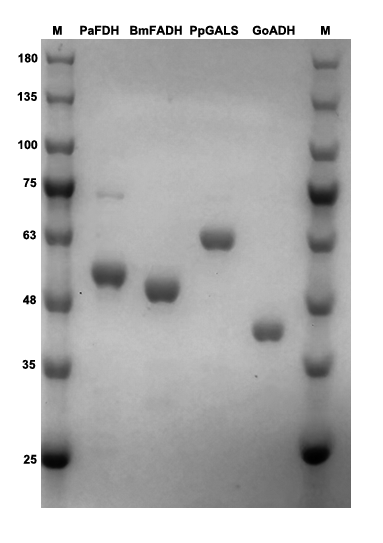


**Figure S4.** SDS-PAGE of purified PaFDH, BmFADH, PpGALS and GoADH. Lane M: protein marker; lane 2: PaFDH; lane 3: BmFADH; lane4: PpGALS; lane 5: GoADH.


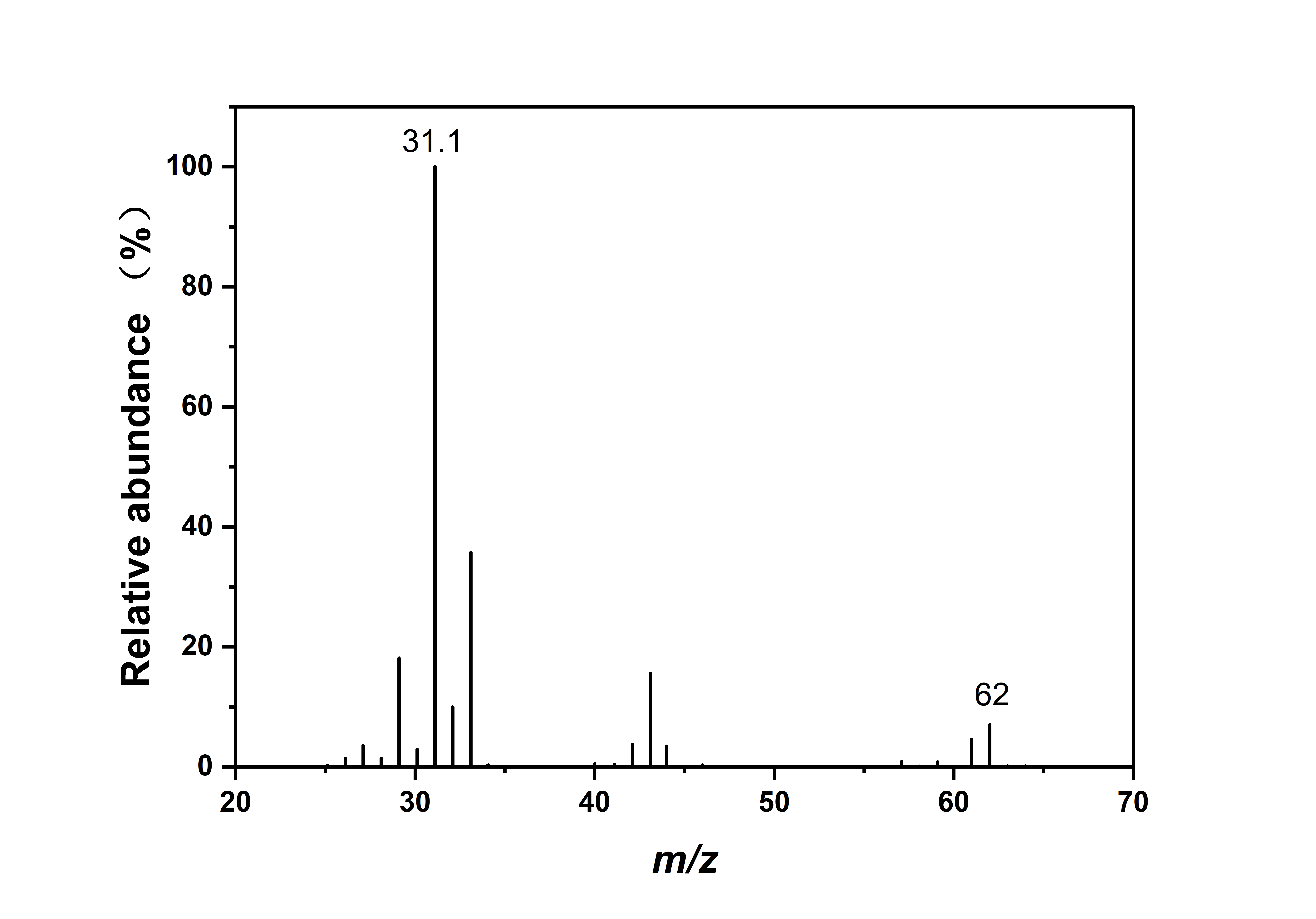


**Figure S5.** GC-MS signal extract of the ethylene glycol in enzymatic reaction mixture.


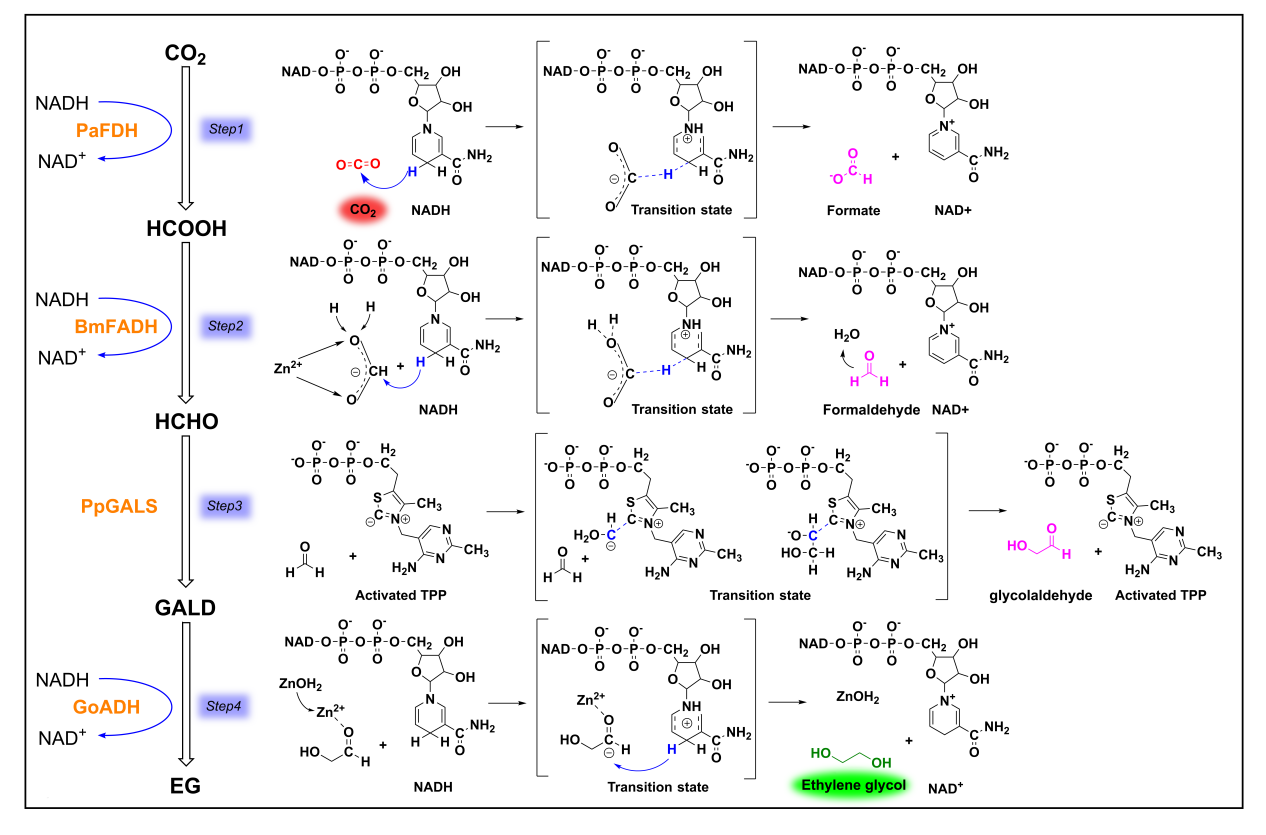


**Figure S6.** Proposed mechanism of PaFDH, BmFADH, PpGALS and GoADH.


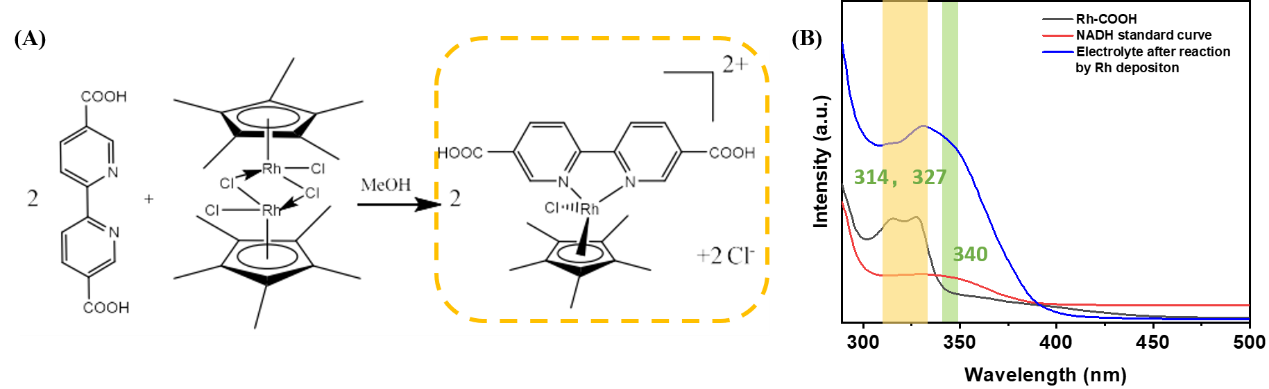


**Figure S7.** (A) Synthesis illustration of Rh complex M.^1^ (B) UV spectra of electro-chemical NADH regeneration reaction mixture by Rh@CF electrode.


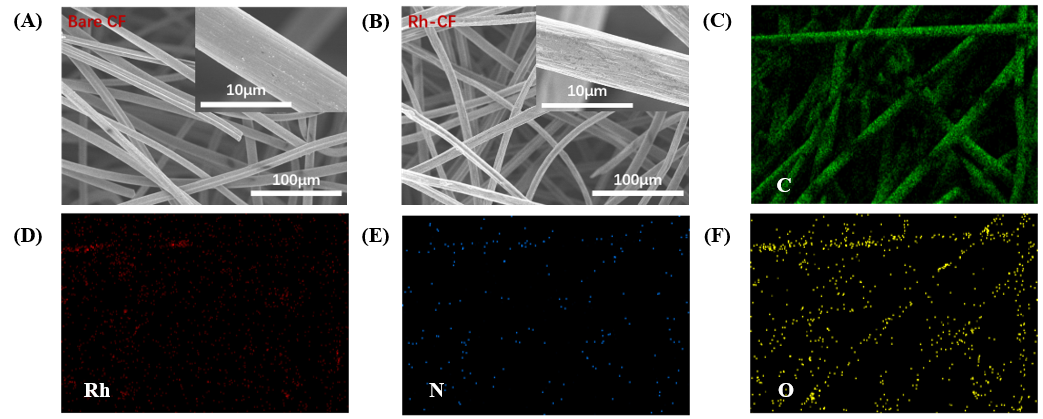


**Figure S8.** SEM images of bare carbon fiber (A) and Rh-CF (B). EDS mapping of Rh-CF for C (C), Rh (D), N (E) and O (F).


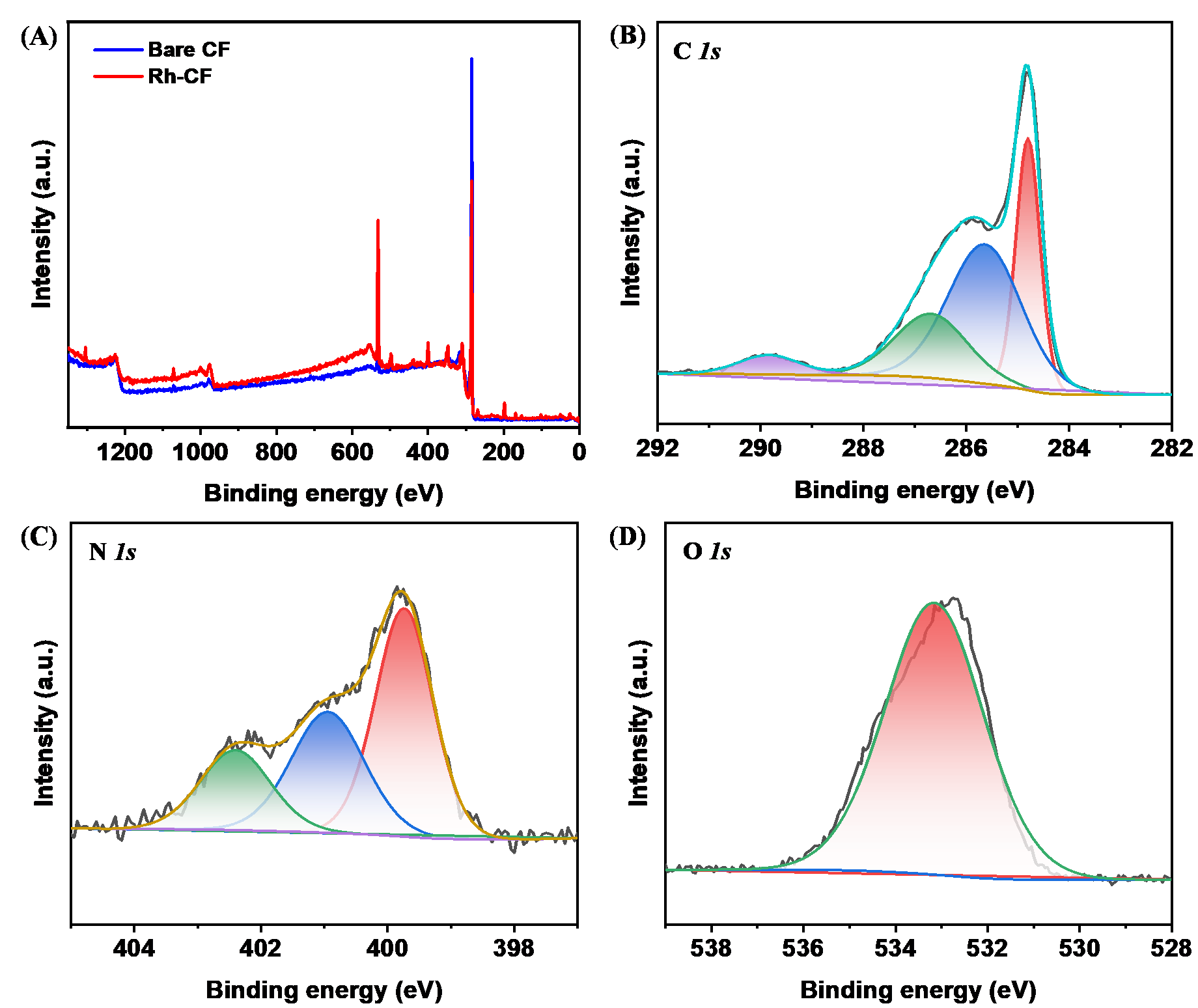


**Figure S9.** (A) XPS survey spectra of bare CF and Rh-CF. (B) XPS spectra of C 1s (B), N 1s (C) and Rh 3d (D) of Rh-CF.


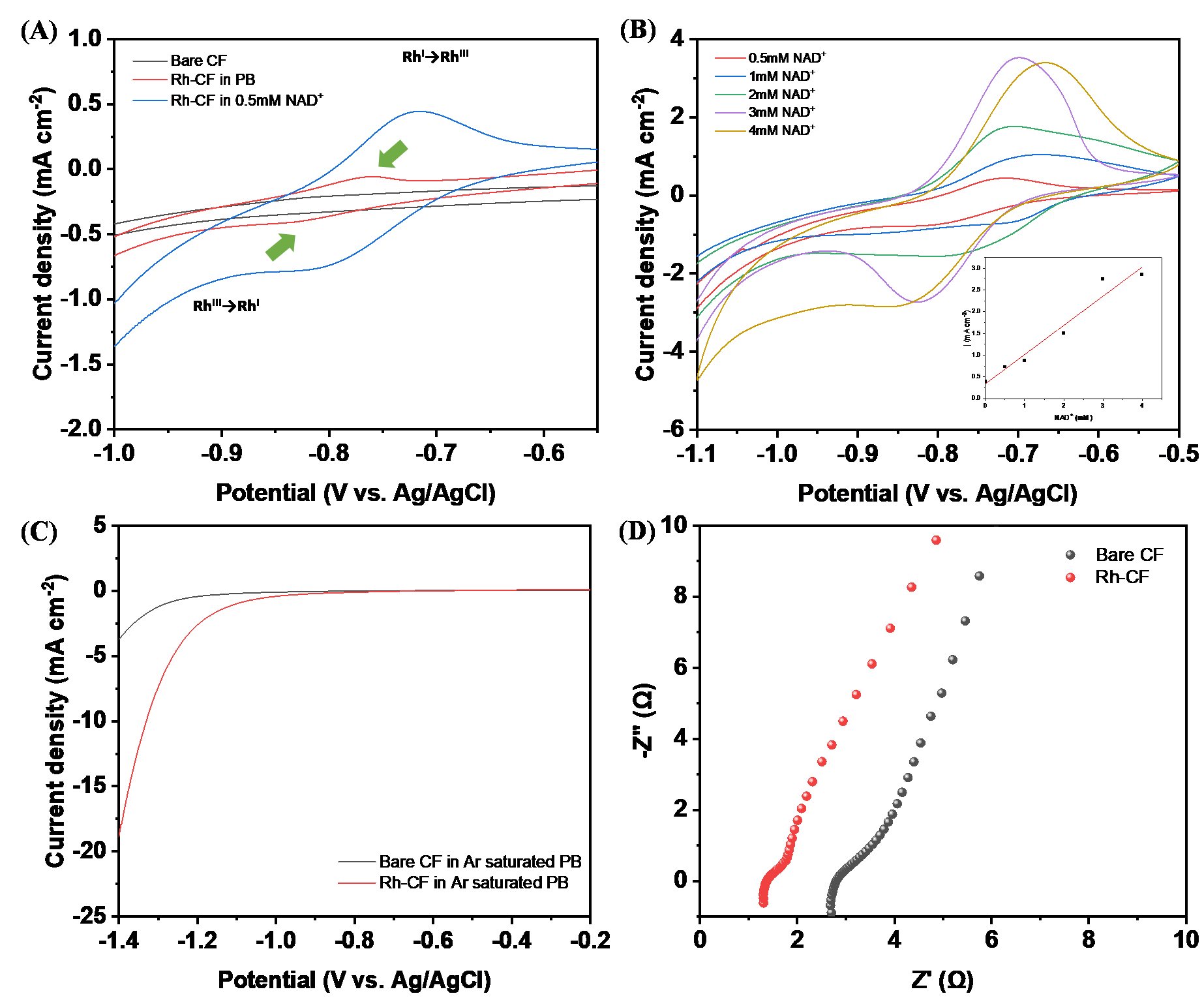


**Figure S10.** (A) CV curves of bare CF in PB, Rh-CF in PB with and without NAD^+^ in PB. (B) CV curves of Rh-CF in different NAD^+^ concentration. (C) LSV of bare CF and Rh-CF in Ar saturated PB. (D) EIS Nyquist plots for bare CF and Rh-CF.

**Figure S11.** NADH yield of Rh-CF with and without applied potential. The applied poteneial was -1.0V *vs.* Ag/AgCl.


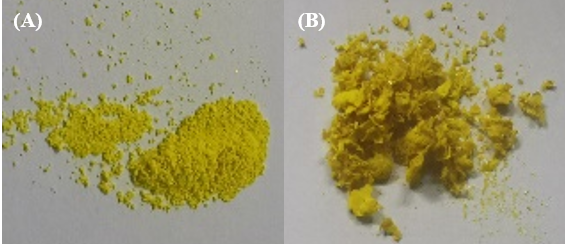


**Figure S12.** Digital images of HOF-1 (A) and ADH@HOF-1 (B).


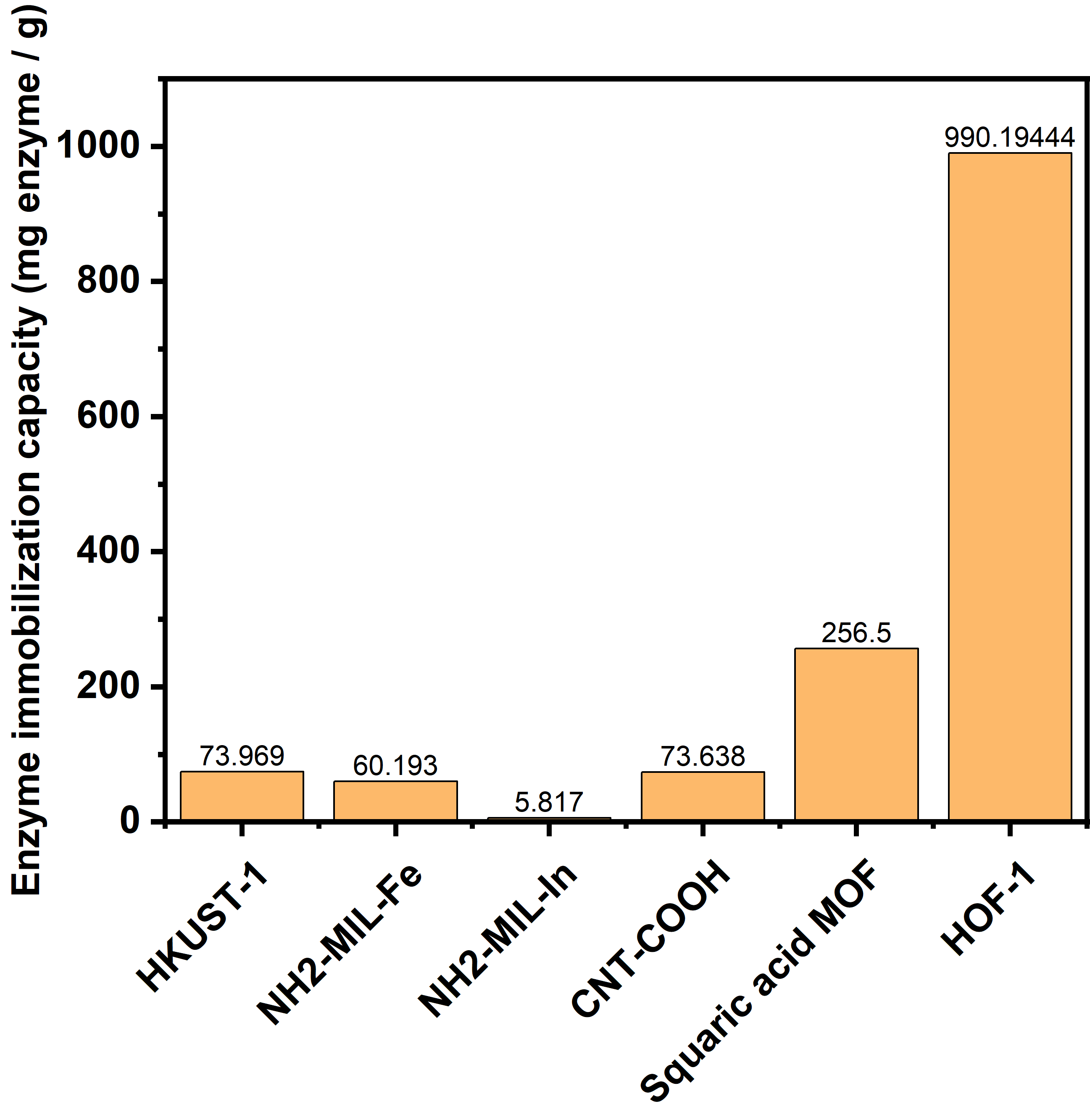


**Figure S13.** Comparison of enzyme immobilization capacity between different materials.


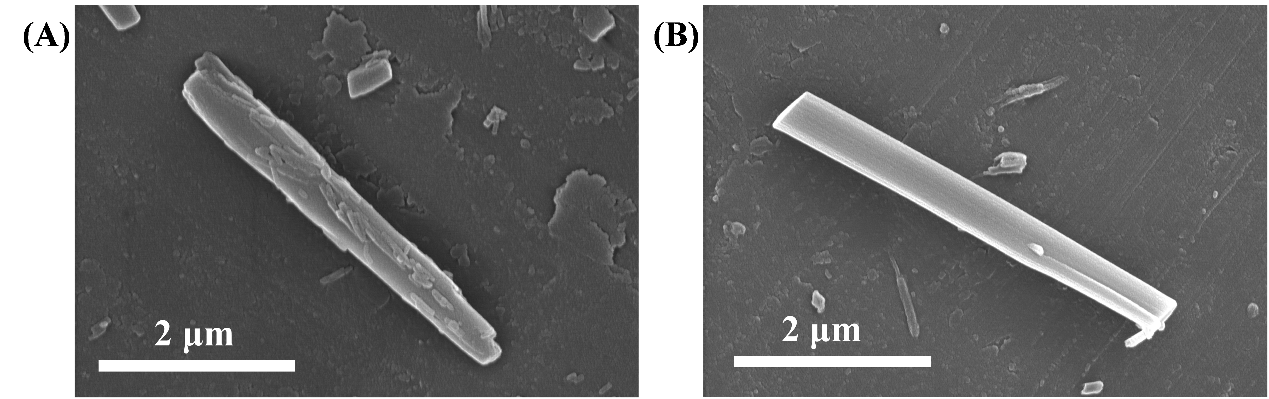


**Figure S14.** SEM images of HOF-1(A) and ADH@HOF-1(B).


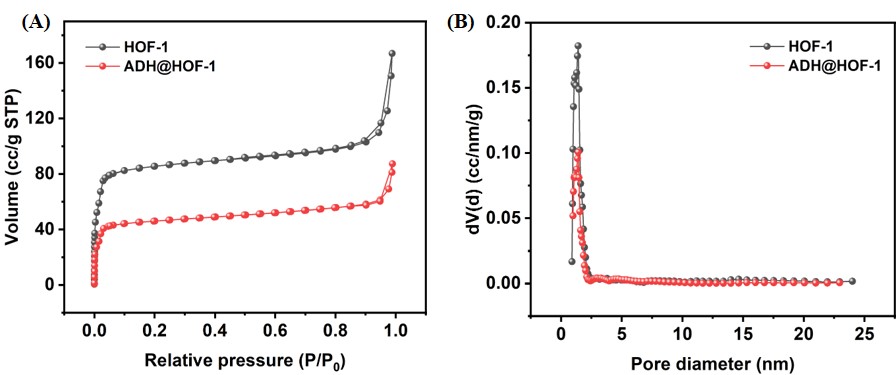


**Figure S15.** (A) N_2_ sorption isotherms for HOF-1 and ADH@HOF-1. (B) Pore distribution of HOF-1 and ADH@HOF-1.


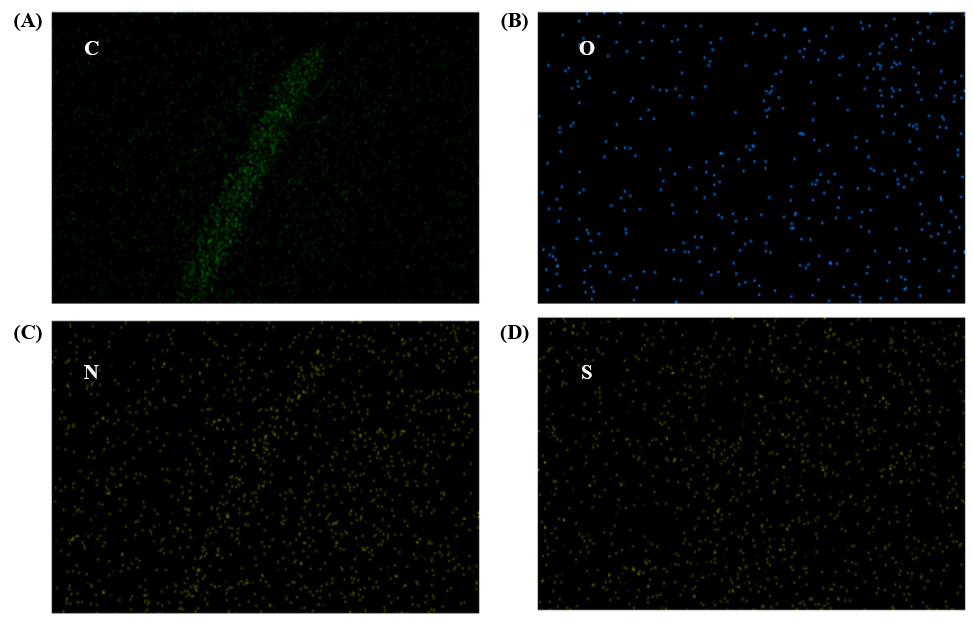


**Figure S16.** EDS mapping of ADH@HOF-1 for C (A), O (B), N (C) and S (D).


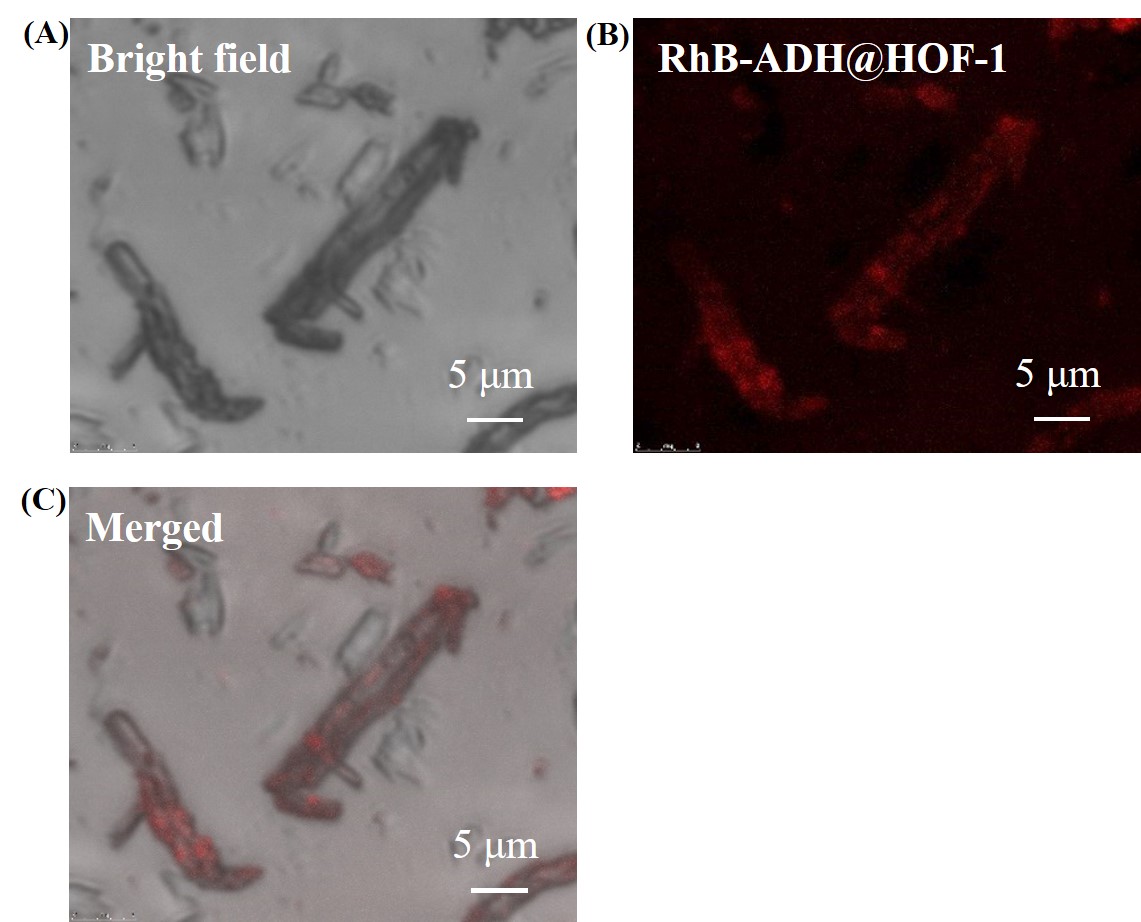


**Figure S17.** CLSM images of Rhodamine B labelled ADH@HOF-1 in bright field (A), dark field (B) and merged field (C).


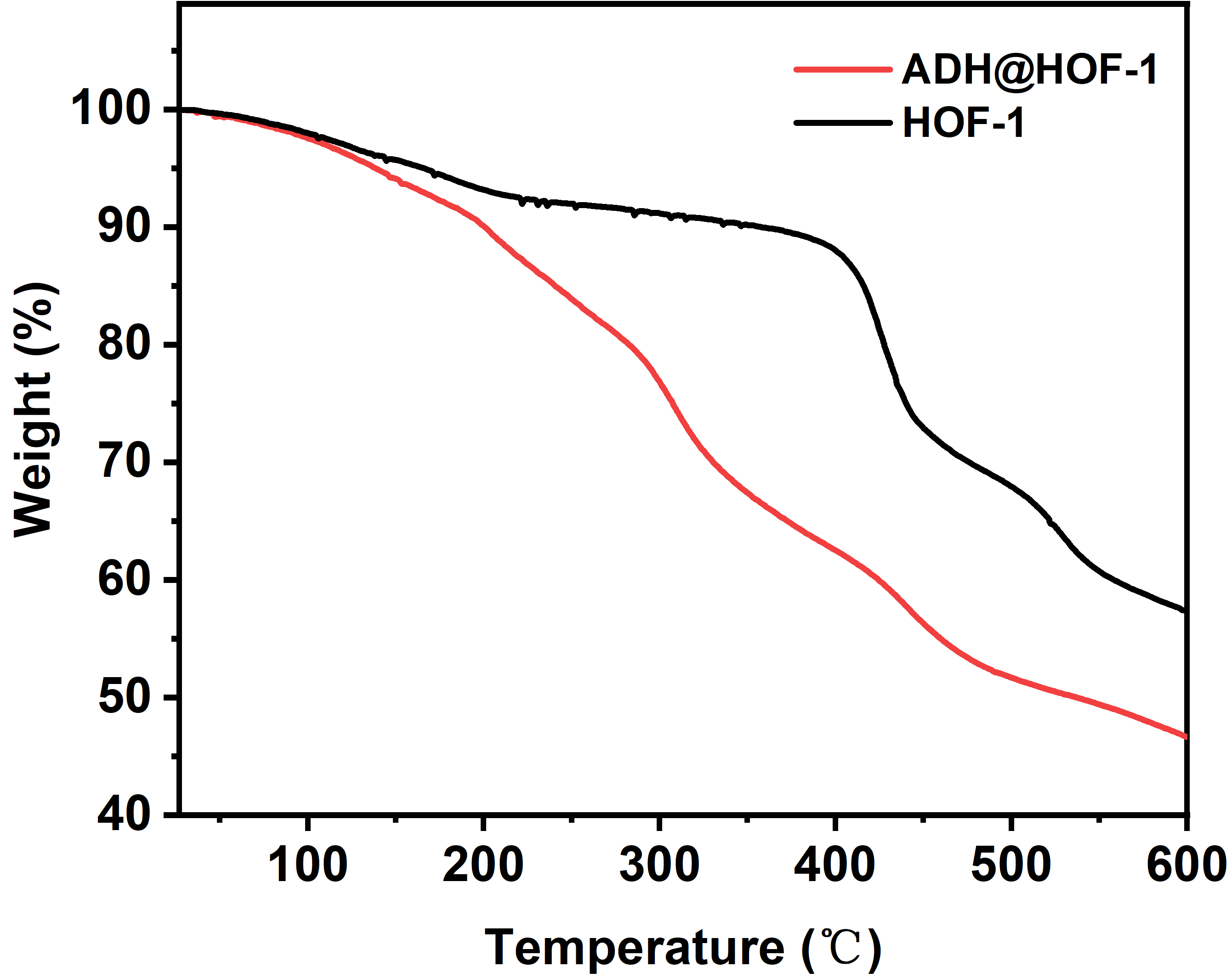


**Figure S18.** TGA profiles of ADH@HOF-1 and HOF-1.


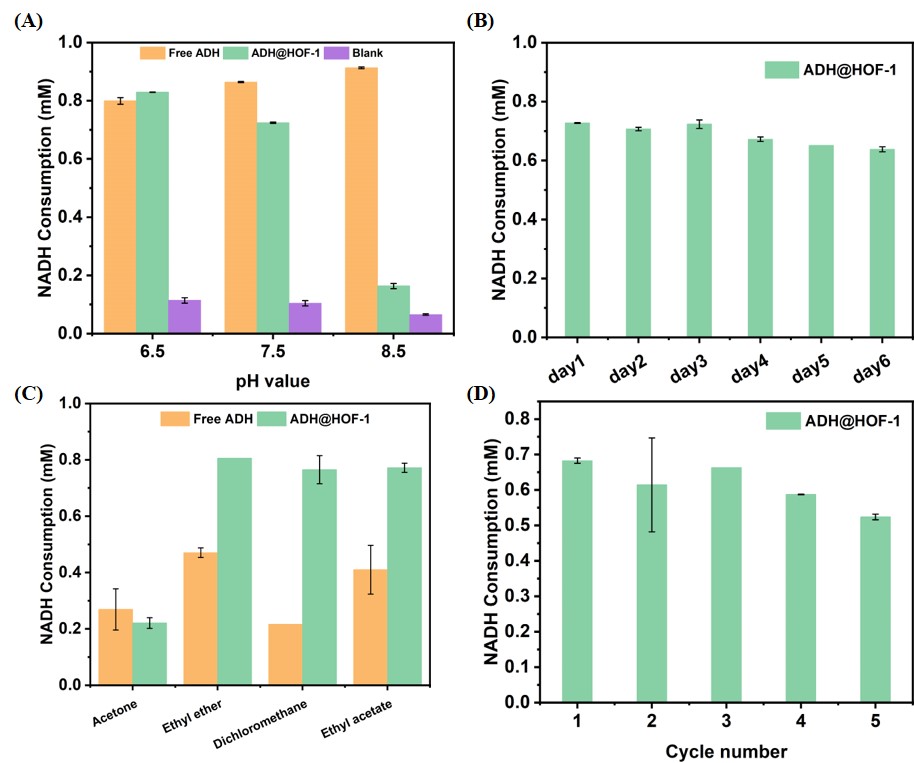


**Figure S19.** (A) Activity of free ADH and ADH@HOF-1 at different pH. (B) Storage stability of ADH@HOF-1. (C) Activity of free ADH and ADH@HOF-1 in organic solvents. (D) Cycle stability of ADH@HOF-1.


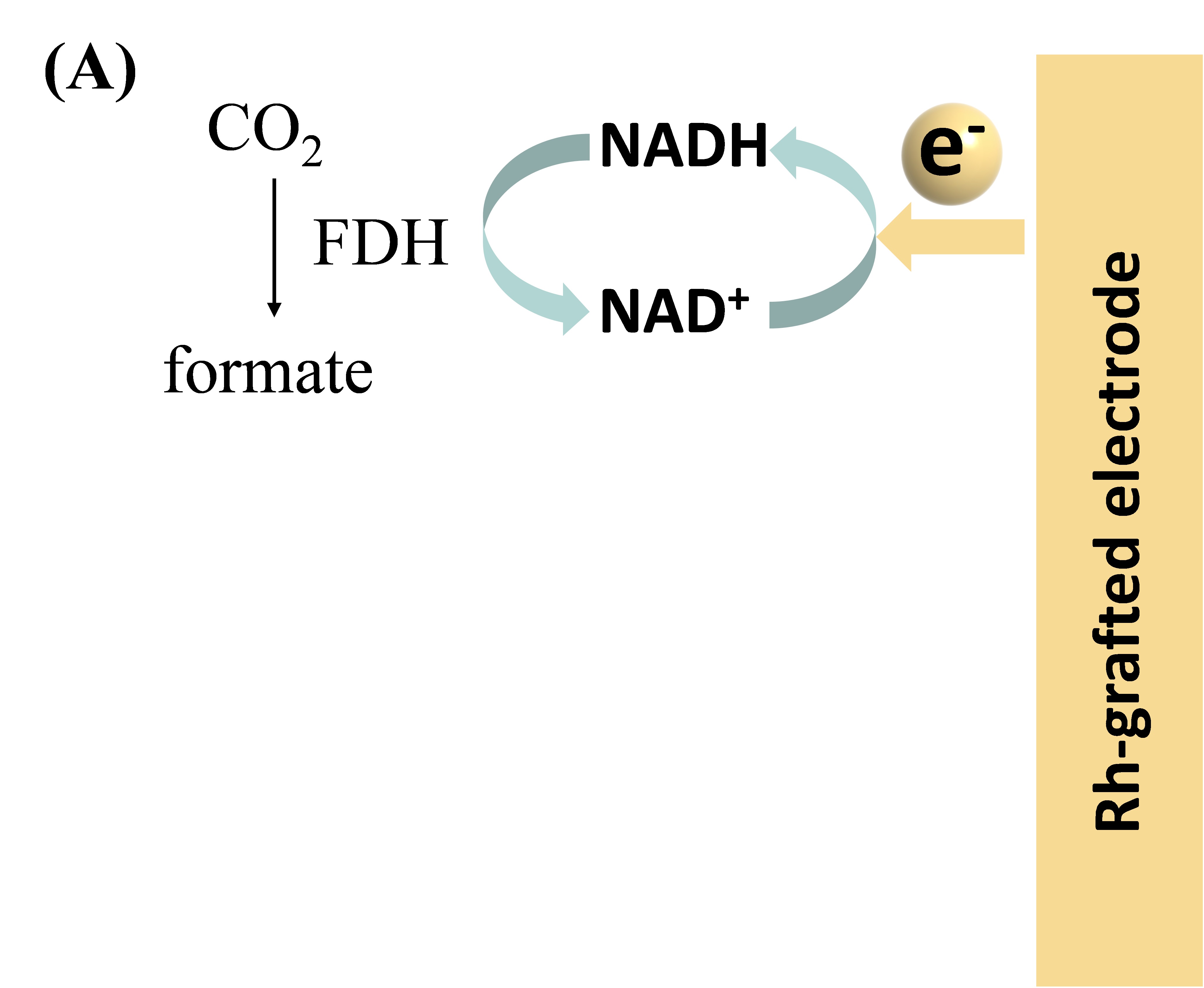

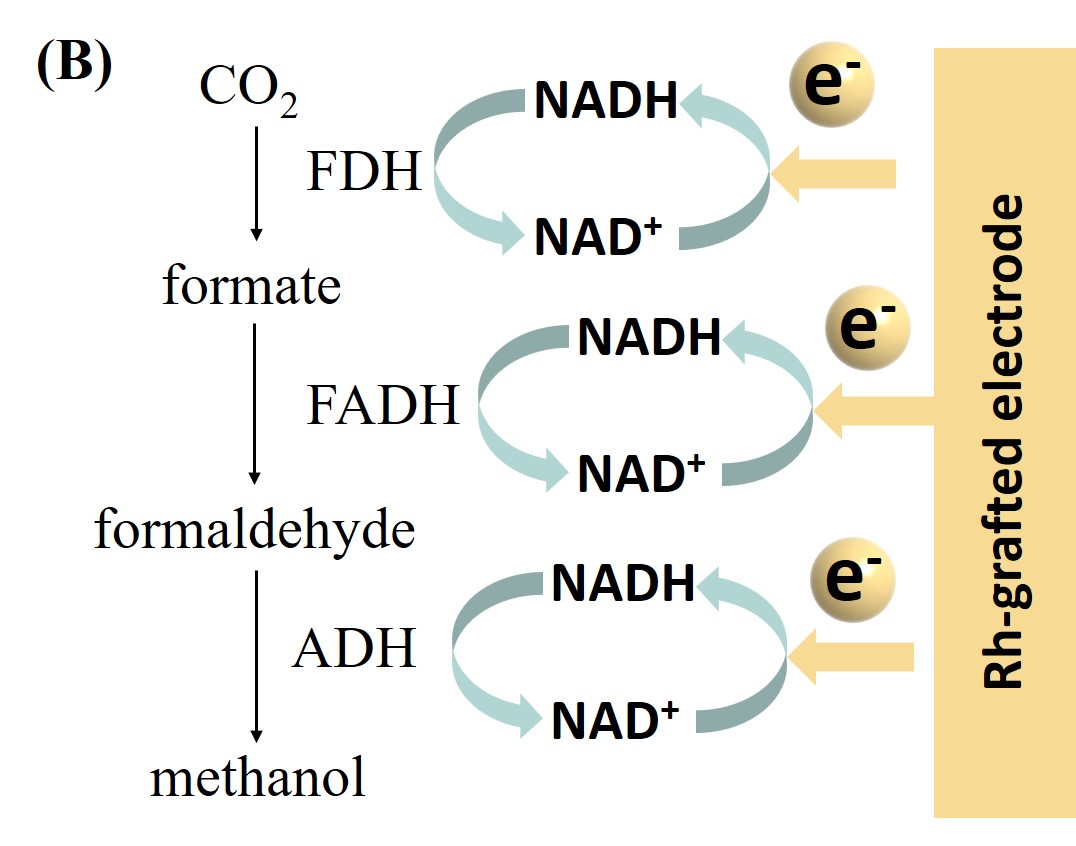


**Figure S20.** Scheme illustration of formate and methanol production separately. It’s worth to emphasize that FDH@HOF and FDH/FADH/ADH@HOF were prepared separately to produce formate and methanol.


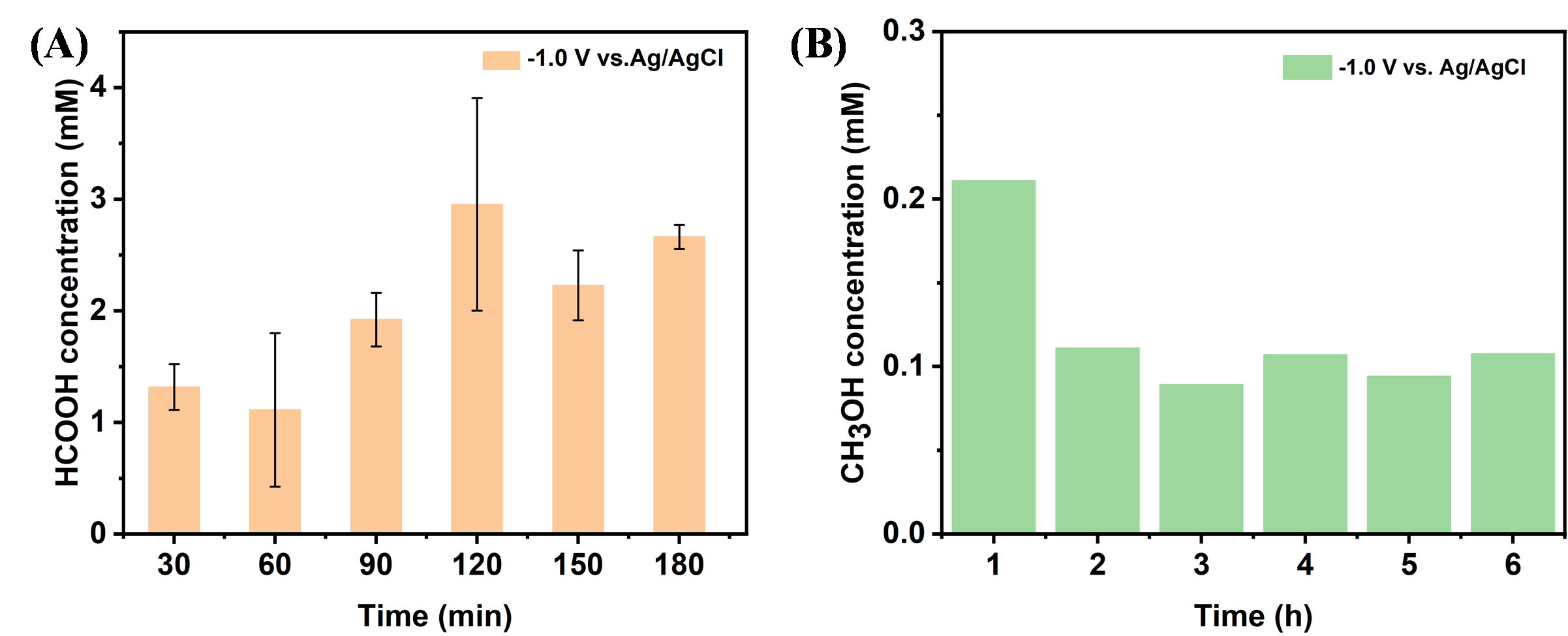


**Figure S21.** HCOOH and CH_3_OH concentration generated by electro-enzymatic catalysis system.


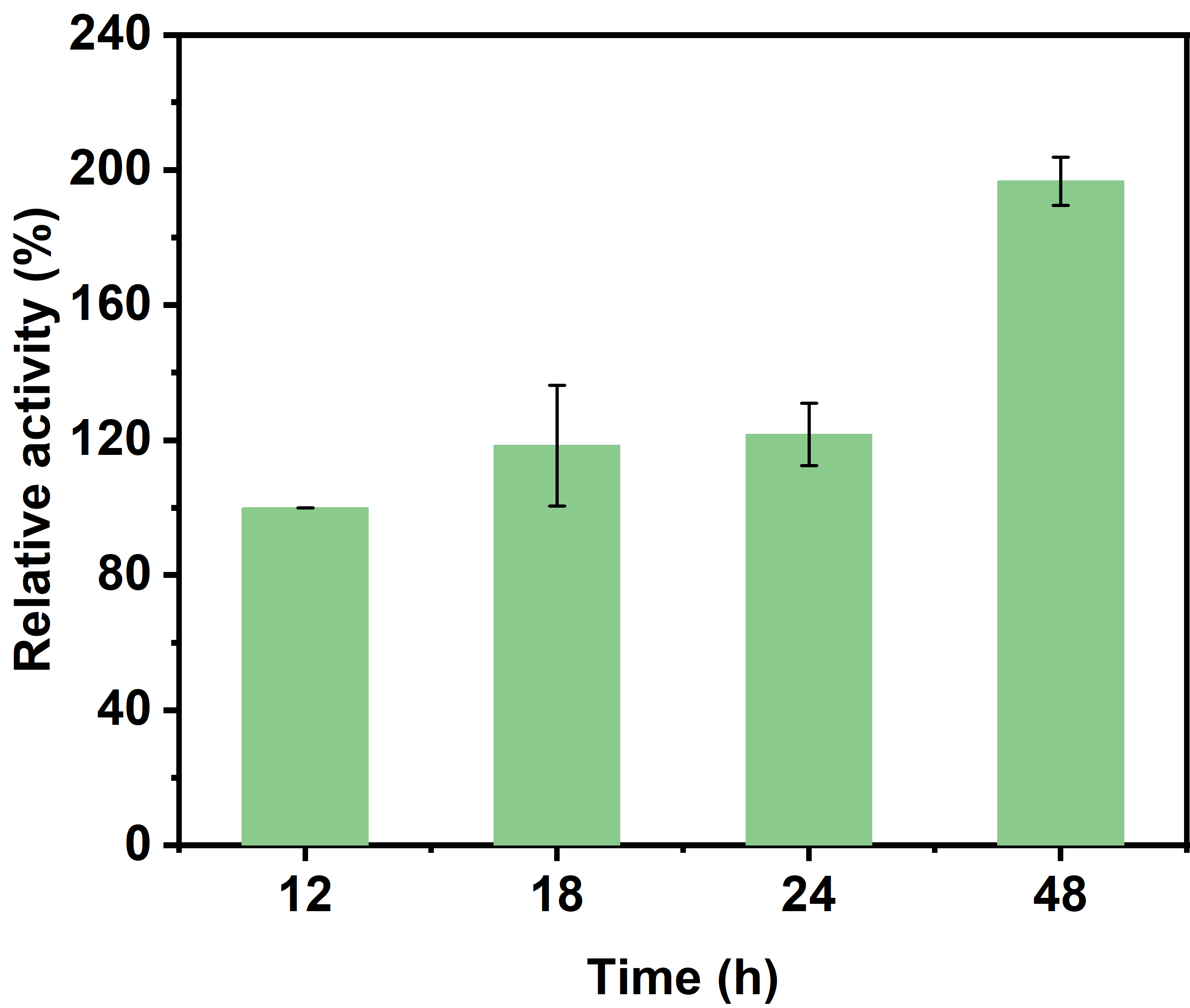


**Figure S22.** Long time activity of enzyme@HOF nano reactor.


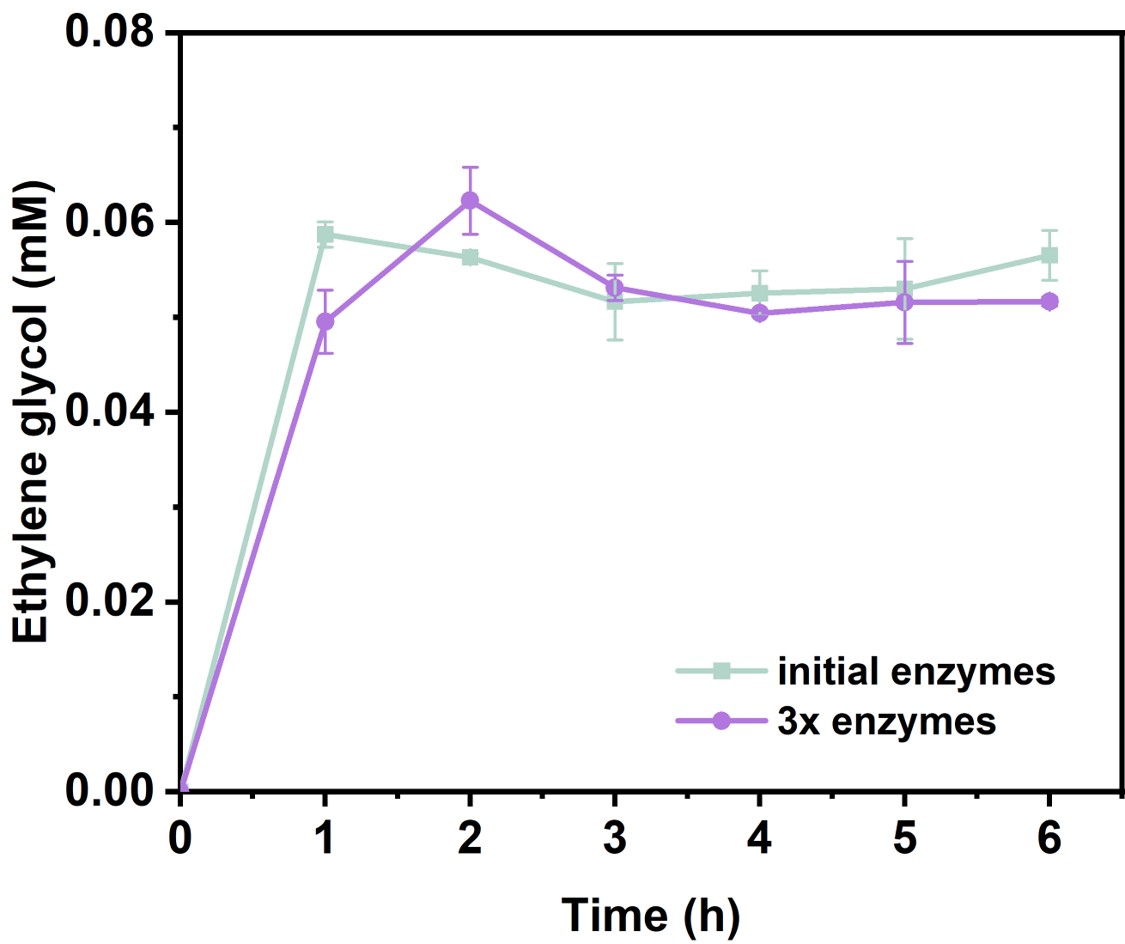


**Figure S23.** When 3 times the amount of intial enzymes were added, there was no obvious improvement in EG yield.


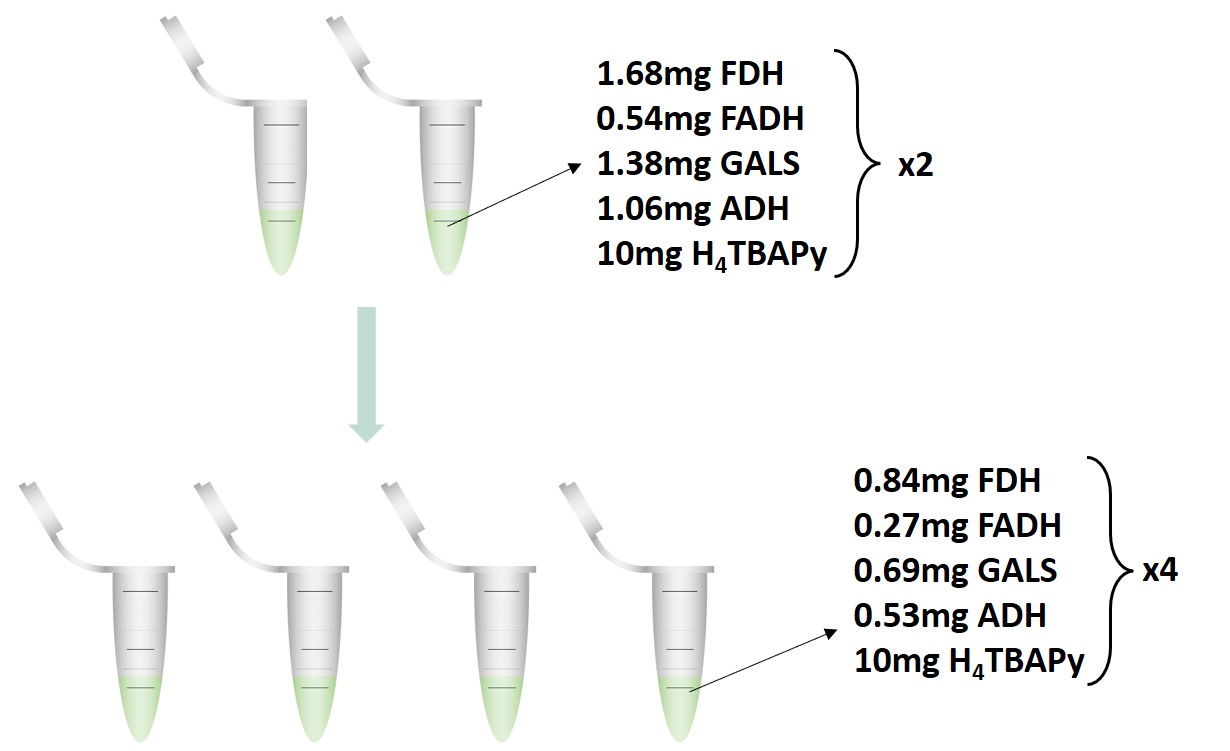


**Figure S24.** Scheme illustration of two different enzyme immobilization method.


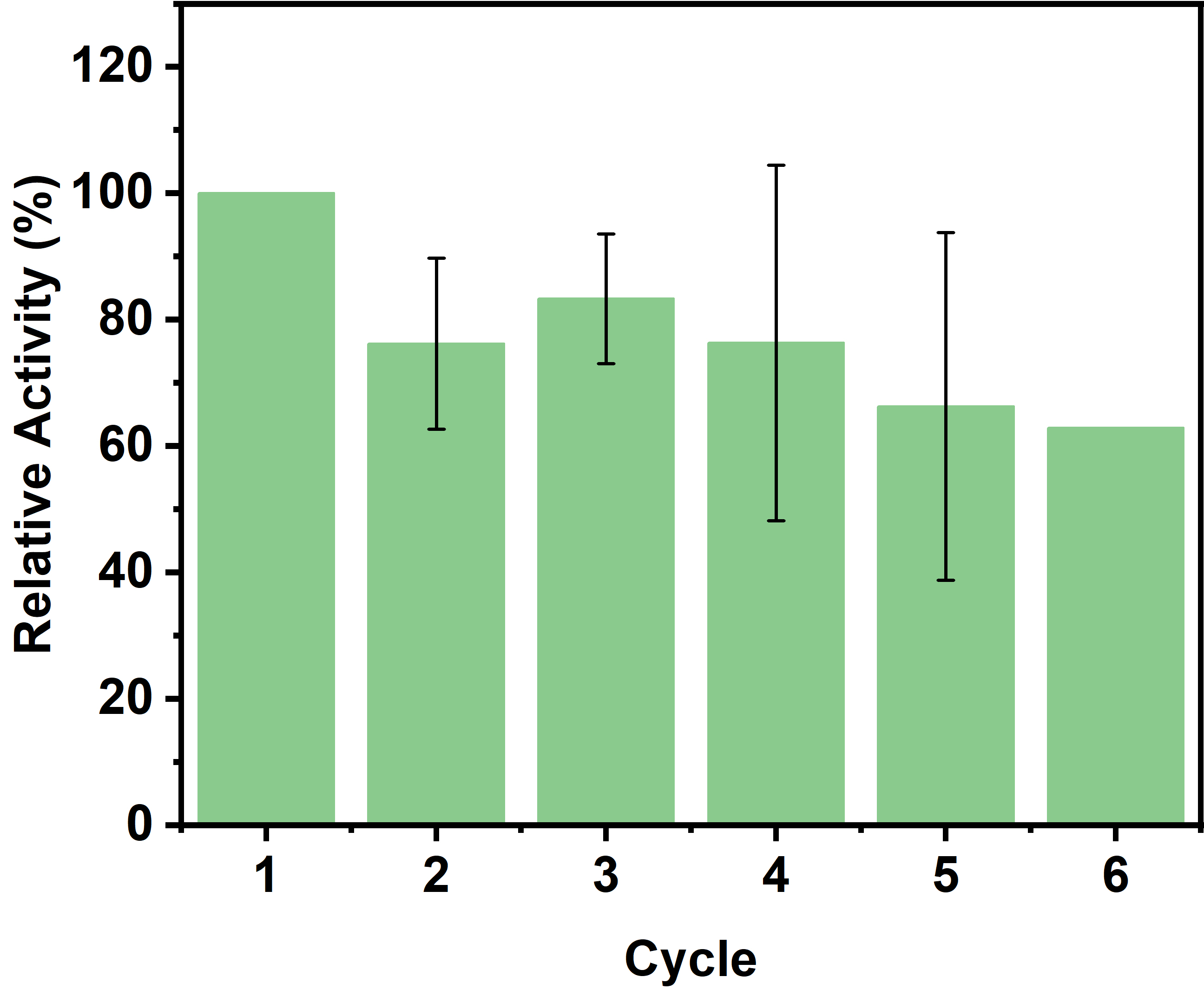


**Figure S25.** Cycle stability of Rh-CF electrode.


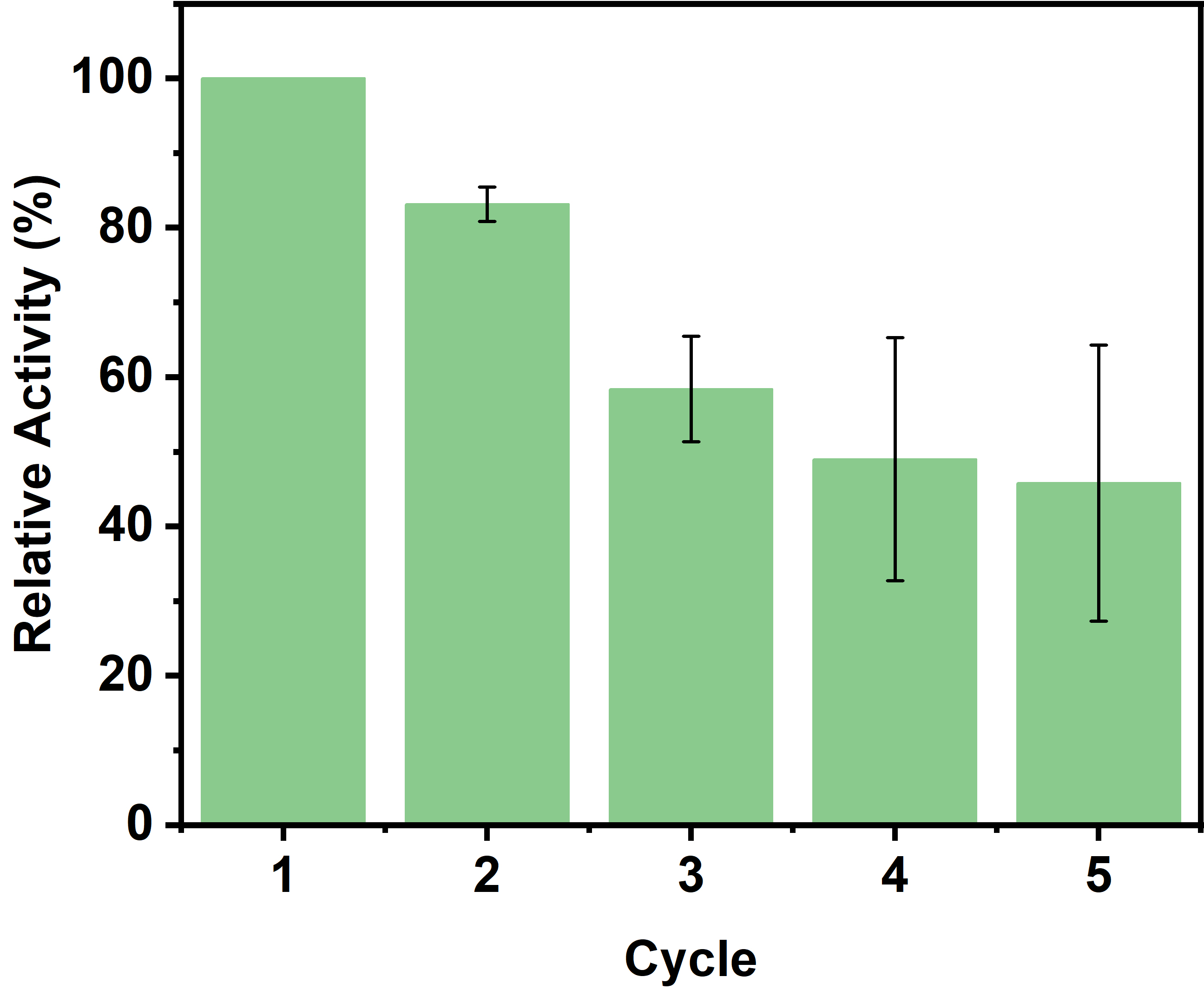


**Figure S26.** Cycle stability of enzyme@HOF nanoreactor.


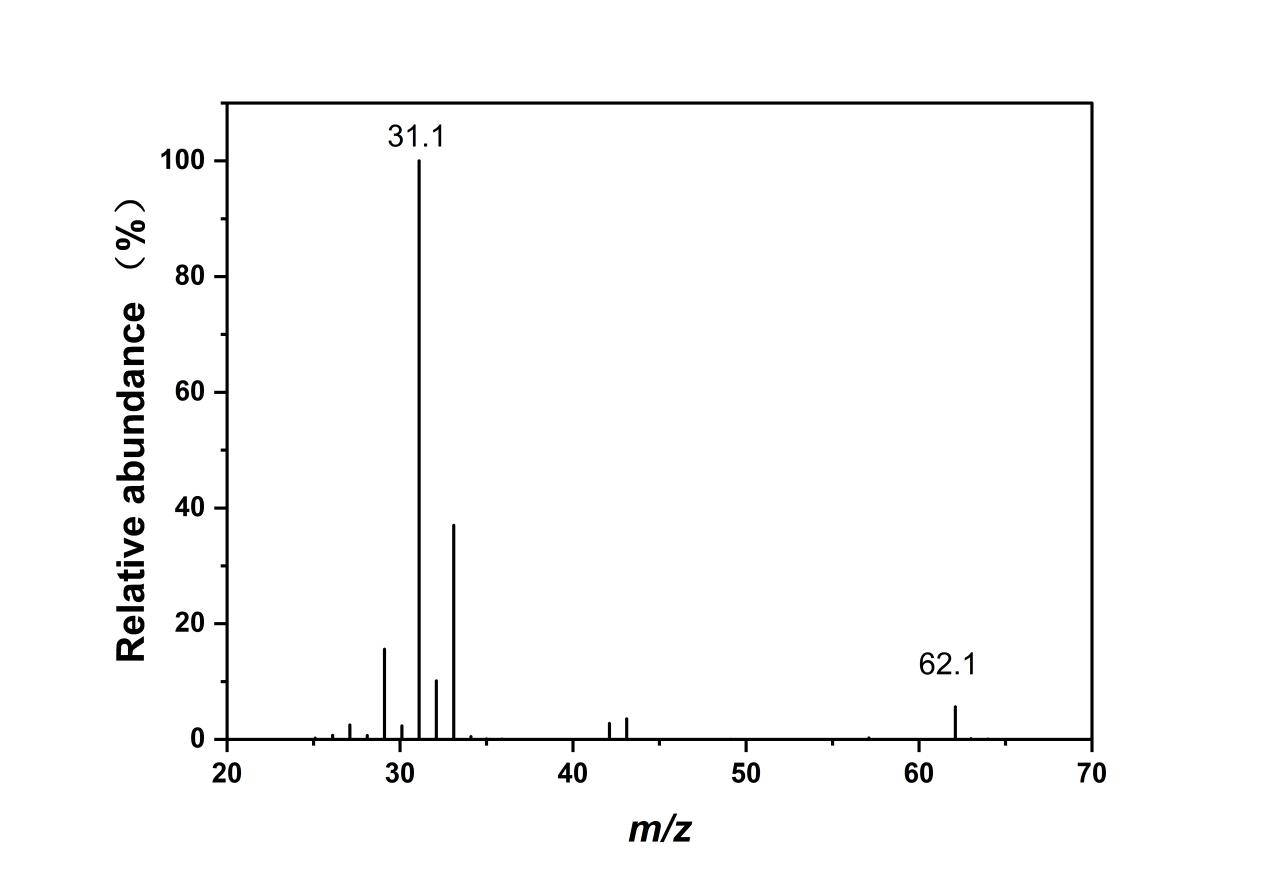


**Figure S27.** GC-MS results of electro-enzymatic EG synthesis.

**Figure S28.** Standard curve of protein concentration.

**Figure S29.** Standard curve of NADH.

**Figure S30.** Standard curve of formate.

**Figure S31.** Standard curve of methanol.

**Figure S32.** Standard curve of 9G.

Table Section

**Table S1** The Gibbs free energy change in each step of the enzymatic cascade pathway for converting CO_2_ to ethylene glycol

| Reaction step | ΔrG' (kJ/mol) | |
| --- | --- | --- |
|  | 1mM NADH | 25mM NADH |
| 1 | 27.6 | 8.6 |
| 2 | 129 | 17.2 |
| 3 | 100.3 | 10.9 |
| 4 | 78.8 | -23.6 |

**Table S2** Comparison of NADH regeneration efficiency between Rh-CF and other Rh based electrodes ^a^

| Catalyst | Immobilization | TOF | Ref. |
| --- | --- | --- | --- |
| [Cp*Rh(bpy)Cl]^+^ | yes | 20 h^−1^ | ^2^ |
| [Cp*Rh(phen)Cl]^+^ | yes | 57 h^−1^ | ^3^ |
| [Cp*Rh(bpy)Cl]^+^ | yes | 3.6 s^−1^ | ^4^ |
| [Cp*Rh(bpy)Cl]^+^ | yes | 164 h^−1^ | ^5^ |
| [Cp*Rh(bpy)H_2_O]^2+^ | no | 0.68 h^−1^ | ^6^ |
| [Cp*Rh(phen)Cl]^+^ | no | 3.5 h^−1^ | ^3^ |
| Cholesterol-modified gold amalgam electrode | yes | 67 h^−1^ | ^7^ |
| [Cp*Rh(bpy)Cl]^+^ | yes | 137.8 h^-1^ | This work |

^a^ Cp=pentamethyl cyclopentadienyl; Rh=Rhodium; bpy=bipyridine; phen=1, 10-phenanthroline.

# References

(1) Zhang, Z.; Li, J.; Ji, M.; Liu, Y.; Wang, N.; Zhang, X.; Zhang, S.; Ji, X. Encapsulation of multiple enzymes in a metal–organic framework with enhanced electro-enzymatic reduction of CO2 to methanol. *Green Chemistry* **2021**, *23* (6), 2362-2371. DOI: 10.1039/d1gc00241d.

(2) Eva Höfer, E. S., Brigitte Ramos, William R. Heineman Polymer-modified electrodes with pendant [RhIII(C5Me5)(L)Cl]+-complexes formed by γ-irradiation cross-linking. *Journal of Electroanalytical Chemistry* **1996**, *402*, 115-122. DOI: 10.1016/0022-0728(95)04243-1.

(3) Lee, J. S.; Lee, S. H.; Kim, J.; Park, C. B. Graphene–Rh-complex hydrogels for boosting redox biocatalysis. *J. Mater. Chem. A* **2013**, *1* (4), 1040-1044. DOI: 10.1039/c2ta00358a.

(4) Tan, B.; Hickey, D. P.; Milton, R. D.; Giroud, F.; Minteer, S. D. Regeneration of the NADH Cofactor by a Rhodium Complex Immobilized on Multi-Walled Carbon Nanotubes. *Journal of The Electrochemical Society* **2014**, *162* (3), H102-H107. DOI: 10.1149/2.0111503jes.

(5) Zhang, L.; Vilà, N.; Kohring, G.-W.; Walcarius, A.; Etienne, M. Covalent Immobilization of (2,2′-Bipyridyl) (Pentamethylcyclopentadienyl)-Rhodium Complex on a Porous Carbon Electrode for Efficient Electrocatalytic NADH Regeneration. *ACS Catalysis* **2017**, *7* (7), 4386-4394. DOI: 10.1021/acscatal.7b00128.

(6) Lee, S.; Choe, H.; Cho, D. H.; Yoon, S. H.; Won, K.; Kim, Y. H. Communication—Highly Efficient Electroenzymatic NADH Regeneration by an Electron-Relay Flavoenzyme. *Journal of The Electrochemical Society* **2016**, *163* (5), G50-G52. DOI: 10.1149/2.0131606jes.

(7) Baik, S. H.; ChanKang; Jeon, C.; EokYun, S. Direct electrochemical regeneration ofNADH from NAD+ using cholesterol-modified gold amalgam electrode. *Biotechnology Techniques* **1999**, *13*, 1-5. DOI: 10.1023/A:1008865212773.
